# Discovery of Antimicrobial Lysins from the “Dark Matter” of Uncharacterized Phages Using Artificial Intelligence

**DOI:** 10.1002/advs.202404049

**Published:** 2024-06-20

**Authors:** Yue Zhang, Runze Li, Geng Zou, Yating Guo, Renwei Wu, Yang Zhou, Huanchun Chen, Rui Zhou, Rob Lavigne, Phillip J. Bergen, Jian Li, Jinquan Li

**Affiliations:** ^1^ National Key Laboratory of Agricultural Microbiology Key Laboratory of Environment Correlative Dietology College of Biomedicine and Health Shenzhen Institute of Nutrition and Health Huazhong Agricultural University Wuhan 430070 China; ^2^ Hubei Hongshan Laboratory College of Food Science and Technology Huazhong Agricultural University Wuhan 430070 China; ^3^ College of Veterinary Medicine Huazhong Agricultural University Wuhan 430070 China; ^4^ Department of Biosystems Laboratory of Gene Technology KU Leuven Leuven 3001 Belgium; ^5^ Monash Biomedicine Discovery Institute Department of Microbiology Faculty of Medicine Nursing and Health Sciences Monash University Melbourne 3800 Australia; ^6^ Shenzhen Branch Guangdong Laboratory for Lingnan Modern Agriculture Genome Analysis Laboratory of the Ministry of Agriculture and Rural Affairs Agricultural Genomics Institute at Shenzhen Chinese Academy of Agricultural Sciences Shenzhen 518000 China

**Keywords:** antibacterial protein, antibiotic resistance, high‐throughput screening, infectious diseases, phage lysin, prophage, stacking model

## Abstract

The rapid rise of antibiotic resistance and slow discovery of new antibiotics have threatened global health. While novel phage lysins have emerged as potential antibacterial agents, experimental screening methods for novel lysins pose significant challenges due to the enormous workload. Here, the first unified software package, namely DeepLysin, is developed to employ artificial intelligence for mining the vast genome reservoirs (“dark matter”) for novel antibacterial phage lysins. Putative lysins are computationally screened from uncharacterized *Staphylococcus aureus* phages and 17 novel lysins are randomly selected for experimental validation. Seven candidates exhibit excellent in vitro antibacterial activity, with LLysSA9 exceeding that of the best‐in‐class alternative. The efficacy of LLysSA9 is further demonstrated in mouse bloodstream and wound infection models. Therefore, this study demonstrates the potential of integrating computational and experimental approaches to expedite the discovery of new antibacterial proteins for combating increasing antimicrobial resistance.

## Graphical abstract

DeepLysin is the first unified software that employs artificial intelligence to mine novel antibacterial proteins from the “dark matter.” The best‐in‐class antibacterial protein LLysSA9 is identified, which can effectively kill drug‐resistant bacteria in vitro and in vivo, being a potent candidate with clinical application prospects. This software expedites the discovery of new antibacterial proteins for combating increasing antimicrobial resistance.

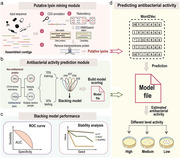

## 1 Introduction

Over the past few decades, the world has relied on antibiotics to treat bacterial infections in humans, animals, and even plants. However, the continued emergence of drug‐resistant pathogens directly resulted in the deaths of at least 1.27 million people globally in 2019.^[^ ^1^ ^]^ Without efforts to curtail resistance or develop new antibiotics, this figure is expected to rise to 10 million deaths annually by 2050, exceeding the number of deaths due to cancer.^[^ ^2^ ^]^ Worryingly, as of December 2020, only 43 antibiotics were in clinical trial or pending approval, in contrast to over 1300 anticancer drugs at similar stages of development.^[^ ^3^ ^]^ This situation is even more concerning, considering that approximately three quarters of antibiotics in development are derivates of existing drugs with similar mechanisms of action, while novel mechanisms targeting problematic pathogens are particularly lacking.^[^ ^4^ ^]^ As resistance to antibiotics can rapidly follow their introduction into the clinic, sometimes emerging even before approval for human use is granted,^[^ ^5^ ^]^ there is an urgent need for novel antibacterial agents to prevent a regression to the pre‐antibiotic era.

The long‐term competition and co‐evolution between bacteria and phages have garnered widespread interest in phage lysins, as they damage bacterial cell wall peptidoglycan, leading to rapid lysis and cell death.^[^ ^6^ ^]^ Lysins differ from small‐molecule antibiotics in that they have a lower tendency to induce bacterial resistance, increased potency, and rapid bactericidal effects.^[^ ^7^ ^]^ Typically, lysins consist of at least one enzymatically active domain (EAD) and a cell‐wall binding domain (CBD).^[^ ^8^ ^]^ Originally, lysins were obtained by amplification from characterized phages or by searching for homologs of existing lysins.^[^ ^9^ ^]^ More recently, the modular architecture of lysins has been utilized to construct chimeric lysins through domain shuffling, enabling the design of tailor‐made antimicrobials with desired properties.^[^ ^10^ ^]^ However, the application of such strategies is severely limited given their heavy reliance on experimentally verified lysins, which constitute only a small fraction of lysins found in nature. To date, only five lysins have progressed to clinical trials.^[^ ^11^ ^]^ The first lysin to enter a phase III trial was Exebacase (also termed CF‐301 or PlySs2), intended for the treatment of *Staphylococcus aureus* bacteremia. Unfortunately, that trial failed an interim futility analysis.^[^ ^12^ ^]^ If the potential of antibacterial proteins such as lysins is to be fully realised, novel discovery and translational strategies are urgently needed to facilitate their entry into clinical practice.

The continued emergence of antibiotic resistance has sparked new interest in using artificial intelligence to assist in antibacterial drug mining and design, primarily focusing on small molecule drugs.^[^ ^13^ ^]^ For example, deep learning has been used to identify antimicrobial peptides from the human gut microbiome^[^ ^14^ ^]^ and to predict the antibacterial activity of kinase inhibitors that inhibit the growth of *Escherichia coli*.^[^ ^15^ ^]^ These examples demonstrate that artificial intelligence has effectively achieved autonomous learning of sequence or structural features that can be used to identify small molecules with antibacterial activity. However, for macromolecular protein candidates, several technological challenges remain unexplored. These include understanding the mechanism by which artificial intelligence extracts protein features, determining which protein features should be extracted, and assessing the feasibility of identifying antibacterial protein candidates. Consequently, artificial intelligence has not been effectively utilized to assist with de novo mining of antibacterial protein candidates. Another significant challenge in developing comprehensive and scalable antibacterial protein mining software is to simplify the usage and developmental workflow for users. Conversely, the development of sequencing technology has exponentially increased the availability of metagenomes and genomes of phages and bacteria (prophage).^[^ ^13b^ ^]^ This vast amount of genetic information, which cannot be confidently assigned to known organisms or functions—aka “dark matter”—provides a huge reservoir of genes encoding lysins which merits further study.

We describe here the development of a software program called DeepLysin, which enabled us to determine how artificial intelligence identifies the features of antibacterial protein candidates and efficiently screens potent lysins from “dark matter.” In total, 17 novel lysin candidates were synthesized, with 7 exhibiting antibacterial activity (41.2%). We further demonstrated that LLysSA9 exhibited greater potency than the best‐in‐class alternative and displayed excellent prospects for clinical application in bacterial systemic infections. This novel open science approach assisted by artificial intelligence to screen non‐redundant lysins lays the methodological groundwork for discovering potent antibacterial protein drugs.

## 2 Results

### 2.1 Using a Stacking Model to Develop Mining Software to Identify Potential Lysins

**Figure 1:**
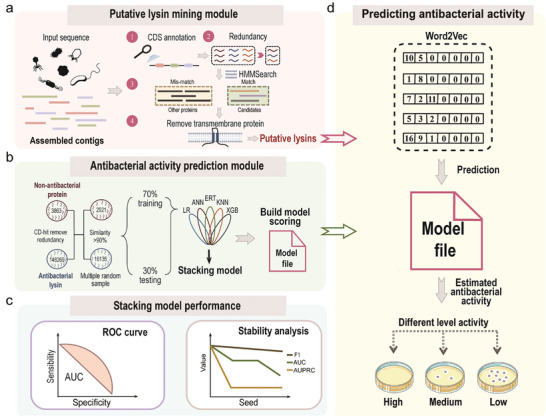
Schematic representation of DeepLysin workflow. a) Mining of putative lysins from assembled contigs. The main steps are CDS annotation, redundancy removal, domain alignment, and transmembrane protein removal. b) The stacking model was employed to train the model in extracting effective features for predicting putative lysin activity. c) The performance of the stacking model was evaluated by F1, MCC, ACC, AUC, and AUPRC metrics; the results for F1, AUC, and AUPRC are shown. d) Predicting putative lysin antibacterial activity.

New tools are urgently needed to fully harness the vast genome reservoirs currently available to researchers, including those capable of efficiently screening for new lysin candidates. To this end, we developed DeepLysin, a step‐by‐step lysin mining software which incorporates two modules (**Figure** **1**). The first module is a lysin mining module that follows a four‐step process (Figure 1a). The first step involves annotating the coding sequences (CDS) of phages and prophages. If the input source is bacterial genomes/metagenomes, prophages are predicted before CDS annotation. Following this, redundant protein sequences are removed to establish a non‐*redundant protein database. Subsequently, HMMsearch is utilized to predict putative lysins within the database. Finally, proteins containing transmembrane domains are excluded to obtain putative lysins*. The core function of this module is to globally explore “dark matter” to narrow down the search space for putative lysins. It can accept input sequences from metagenomes or bacteria (prophage)/phage at any assembly level (contig, scaffold, chromosome, or complete genomes), and provide a comprehensive and scalable service to screen putative lysins with potential activity against any drug‐resistant pathogen. *However, since this module utilizes* traditional blastP/protein sequence alignment‐based methods (Figure 1a), it *exhibited coarse prediction performance*.

**Figure 2:**
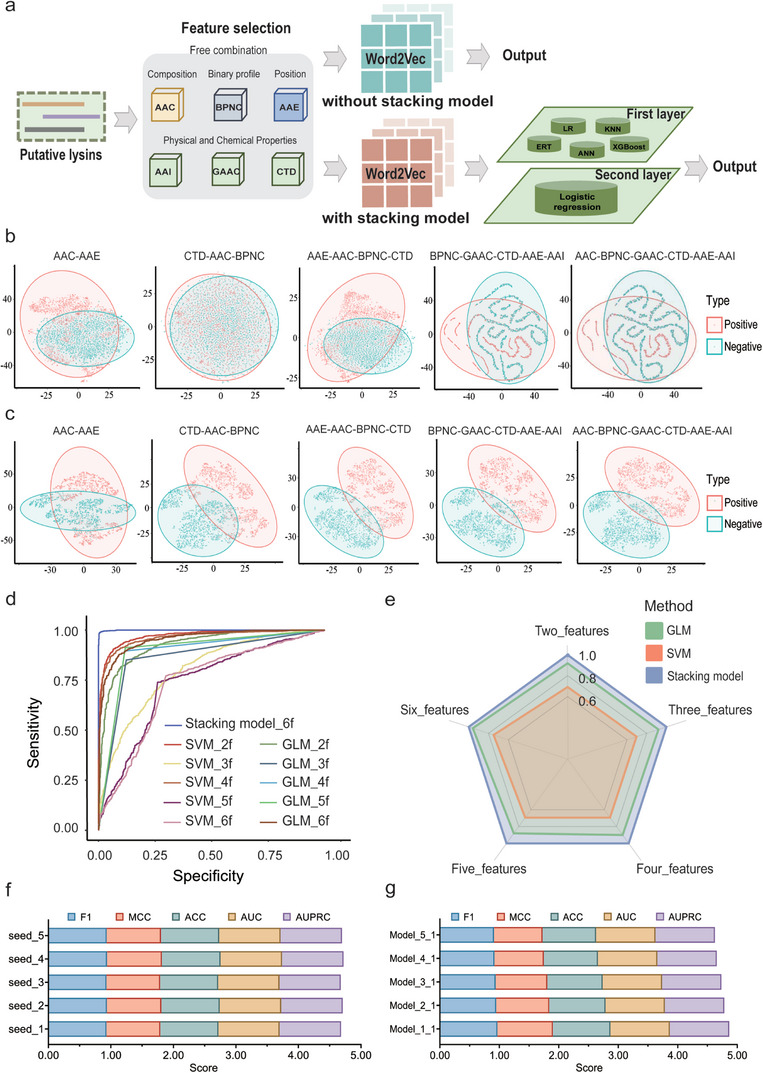
Performance of the stacking model. a) Structure of the stacking model. Six features were provided to capture specific lysin characteristics. The five algorithms used to construct the first layer of the model were ERT, ANN, KNN, XGBoost, and LR. The second layer utilized LR to evaluate antibacterial activity. b, c) 2D t‐SNE visualization of different feature combinations b) without calculation and c) with calculation by the first layer of the stacking model. d) ROC curves of different feature combinations (AAC_AAE, CTD_AAC_BPNC, AAE_AAC_BPNC_CTD, BPNC_GAAC_CTD_AAE_AAI, and AAC_BPNC_GAAC_CTD_AAE_AAI) and various machine learning models (SVM, GLM, and Stacking model) using the training dataset with 5‐fold cross‐validation. e) AUPRC of different feature combinations and various machine learning models (SVM, GLM, and Stacking model) using the training data set with fivefold cross‐validation. f,g) Model performance based on datasets with f) different random seeds of positive samples, and g) ratios of positive and negative samples, with fivefold cross‐validation. F1, MCC, ACC, AUC, and AUPRC scores were obtained from the trained model. For each parameter, higher scores indicate better performance.

The accuracy of active lysin prediction was further improved by the development of a second module, the antibacterial activity prediction module, which *estimates the antibacterial activity of putative lysins identified by the* lysin mining module (Figure 1d *)*. This second module employs *artificial intelligence to autonomously learn to distinguish diverse and* high‐dimensional protein features (Figure 1b *)*, evaluating the performance of different models using five parameters (F1, MCC, ACC, AUC, and AUPRC) (Figure 1c *; details are given in* **Figure** **2**b–g *)*.

*Initially, using only Word2vec to extract* six raw protein features (AAI, AAE, AAC, CTD, BPNC, GAAC), whether individually or in random combinations, was found to be insufficient to effectively distinguish between positive and negative samples (Figure 2b; Figure S1, Supporting Information). Subsequently, *a two‐layer stacking model proved optimal for predicting the activity of putative lysins (*Figure 1b *; details are provided in* Figure 2a *)*. The first layer combines five common machine learning models: ERT (Extremely randomized trees), LR (Logistic regression), ANN (Artificial neural network), KNN (K‐nearest neighbor), and XGBoost (Extreme gradient boosting), to extract the most effective features from the input raw protein sequences. Incorporating this layer dramatically increases discrimination between positive and negative samples after the first layer calculation, with the combination of all six raw protein features having the highest silhouette coefficient (Figure 2c; Figure S2, Supporting Information). A second layer was subsequently introduced to reduce the dimensionality of high‐dimensional features. This layer uses LR as a non‐linear activation function to generate the final activity predictions as scores between 0 and 1, where higher scores indicate greater antibacterial activity.

An important and unique feature of DeepLysin was the ability of the *antibacterial activity evaluation module* to elucidate how artificial intelligence accurately identifies protein features and selects the optimal model for mining antibacterial protein drug candidates. When the same dataset was utilized, the stacking model outperformed other traditional machine learning models (SVM, GLM, etc.) in identifying protein features (AAC, BPNC, AAE, AAI, GAAC, CTD), with an AUC of 0.99 (other methods ranged from 0.73 to 0.95) (Figure 2d) and AUPRC of 0.99 (other methods ranged from 0.68 to 0.96) (Figure 2e; Table S1, Supporting Information). Fluctuations in performance when evaluated by five parameters (F1, MCC, ACC, AUC and AUPRC) showed only negligible differences with multiple random positive sampling (five times) (Figure 2f) and imbalance ratios of positive and negative samples (ranging from 1:1 to 5:1) (Figure 2g). These results demonstrate that the incorporation of the stacking model into DeepLysin produced a robust model for identifying lysins from genome reservoirs. *Collectively, the lysin* mining and *antibacterial activity evaluation modules form a unified software package for lysin identification*.

### 2.2 Identification from “Dark Matter” of Lysin Candidates Against *S. aureus*

**Figure 3:**
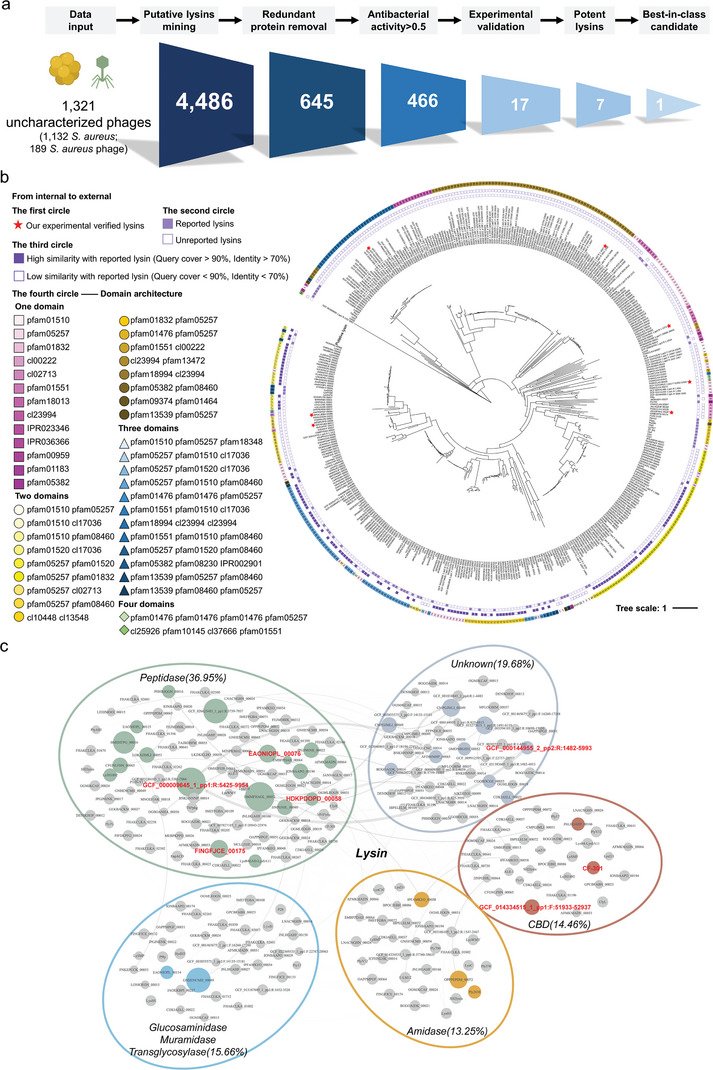
Schematic representation of the procedure for designing *S. aureus* lysins. a) Procedure for screening lysins with activity against *S. aureus*. Data from 1132 *S. aureus* genomes and 189 *S. aureus* phage genomes with the highest annotation level in NCBI were collected and inputted into the lysin mining module. The 4486 proteins predicted contained 645 nonredundant proteins. The antibacterial activity prediction module then identified 466 putative lysins with antibacterial activity exceeding 0.5. Finally, 17 lysins were randomly selected for experimental verification and a best‐in‐class lysin obtained. b) Selection of natural lysins. The phylogenetic tree illustrates the relationship between 424 lysins, including 357 *S. aureus* putative lysins (molecular weight < 80 kDa), and 67 reported lysins. The experimentally verified lysins are shown in the first (inner) circle and are marked by red stars. The second and third circles identify lysins reported in the literature and the similarity of putative lysins with reported lysins, respectively. The fourth circle shows the domain architecture of lysins. c) Selection of chimeric lysins. Network‐based relationships between domains were analyzed, and lysins split according to the domain predicted by pfam. Domains with > 80% similarity were clustered into one node. Nodes are connected by edges, which are directional and represent the order of connection between different domains. The node size represents the possibility of a domain combining with other domains to form a complete lysin, which was called connectivity. Nodes with connectivity > 3 are displayed in color. Seven different domains (shown in bold) were randomly selected for domain shuffling to obtain ten chimeric lysins for experimental verification. Larger versions of (b,c) are shown in Figures S4 and S5, Supporting Information.

*S. aureus* is a Gram‐positive pathogen of global significance. The ability of DeepLysin to successfully mine “dark matter” and identify lysin candidates active against *S. aureus* was assessed by analyzing 1321 uncharacterized phages (1132 *S. aureus* genomes and 189 *S. aureus* phage genomes). Of the 4486 proteins identified, 645 were nonredundant lysins of which 466 were predicted to have antibacterial activity above 0.5. Among these were 357 lysins with a molecular weight < 80 kDa (**Figure** **3**a). All thirty‐three reported lysins from *Staphylococcus* were screened out (Table S2, Supporting Information). Importantly, 63.9% (228/357) of putative lysins shared less than 70% sequence identity with reported lysins (Figure 3b). These results demonstrate DeepLysin's ability to reliably identify novel lysins from “dark matter” for further experimental testing. The putative lysins covered various domains with diverse mechanisms of action, with half containing two predicted functional domains (summarized in Figure S3a–e, Supporting Information).

**Figure 4:**
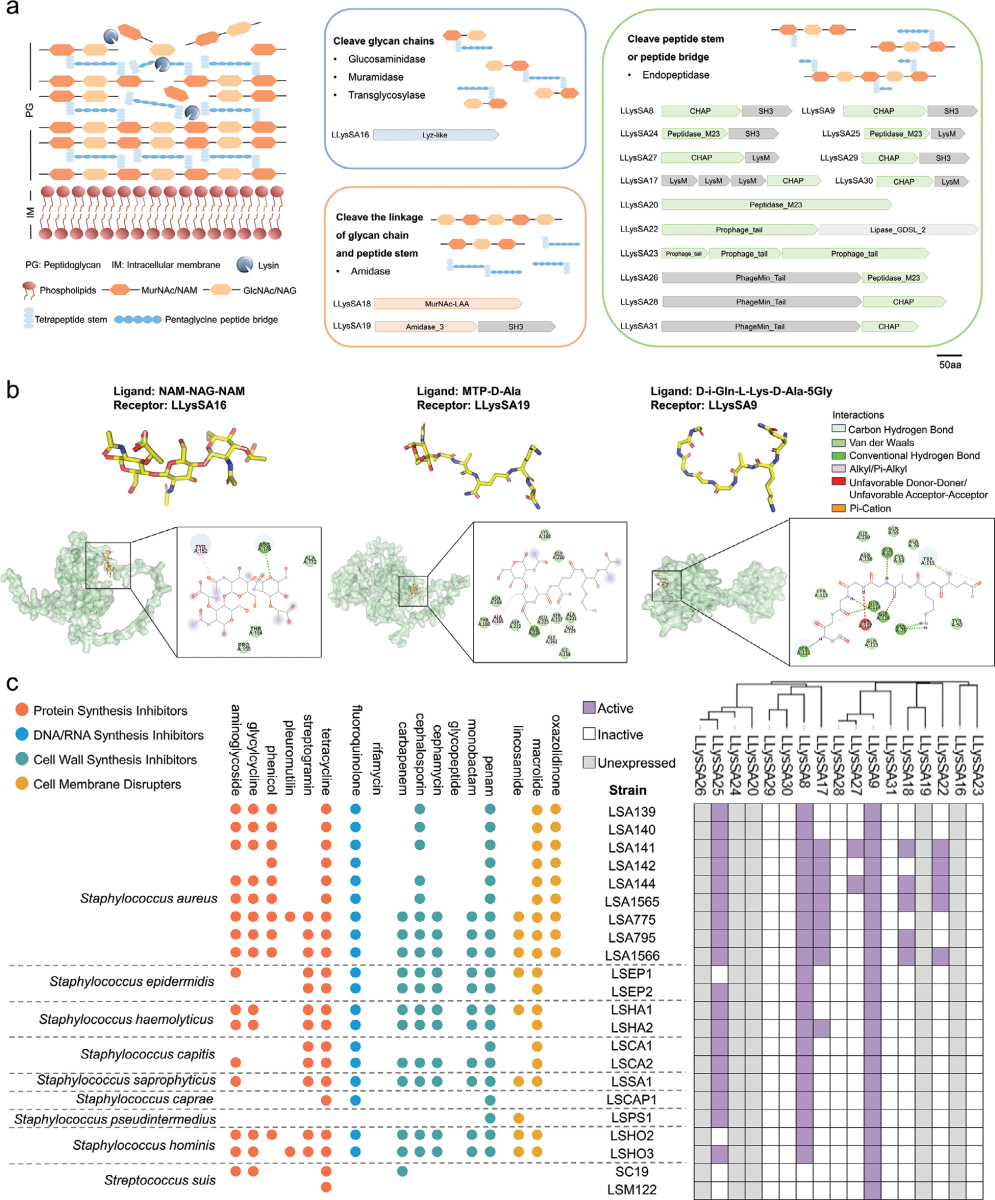
Prediction of lysin‐peptidoglycan interactions and experimental validation of putative lysins. a) Classification and domain architecture of 17 putative lysins. The lysins were classified into three groups according to the predicted peptidoglycan cleavage site. b) Possible lysin‐peptidoglycan interaction sites. The different peptidoglycan fragments were used as ligands according to the cleavage site of putative lysins. The 3D lysin structure was predicted by AlphaFold2 and the putative interactions between lysin (green) and ligand (yellow) by AutoDock. The interactions were visualized by Discovery Studio. NAM, N‐Acetylmuramic acid. NAG, N‐Acetylglucosamine. MTP, N‐acetylmuramyl‐L‐alanyl‐D‐iso‐glutamyl‐L‐lysine. c) In vitro validation of putative lysin activity. The left panel shows antibiotic‐resistance genes (determined by the Comprehensive Antibiotic Resistance Database) present in the 22 strains tested, while the right panel shows the initial screen for antibacterial activity of 17 putative lysins against these strains. Active lysins (i.e., those forming plaques) are shaded purple and inactive lysins are white. Lysins not expressed following bacterial transformation are shaded gray.

To identify novel lysins, a phylogenetic tree was constructed from 424 lysins, which included 357 putative lysins and 67 reported lysins. Seven lysins were randomly selected from different clades lacking experimentally verified lysin homologs for subsequent experimental verification (Figure 3b; Figure S4, Supporting Information). To determine domains with high degrees of connectivity for shuffling and based on our hypothesis that the frequent collocation of EAD and CBD domains increases the probability of generating active chimeric lysins, an EAD‐CBD correlation network was also established using 645 putative lysins and 67 reported lysins. According to the EAD‐CBD correlation network, seven different domains with connectivity higher than three were randomly selected to form ten chimeric lysins for further experimental testing (Figure 3c; Figure S5, Supporting Information). Overall, seventeen putative lysins (seven natural and ten chimeric) were selected for molecular docking studies to preliminarily evaluate the accuracy of DeepLysin in identifying novel lysins. These were classified into three categories based on their predicted peptidoglycan cleavage site (**Figure** **4**a; Tables S3 and S4, Supporting Information). After predicting their 3D structures, the docking studies revealed that the putative lysins interacted with their corresponding peptidoglycan fragments through the formation of hydrogen bonds, van der Waals forces, Pi‐alkyl interactions, etc., suggesting the possibility of peptidoglycan cleavage (Figure 4b; Figure S6, Supporting Information) and confirming the ability of DeepLysin to provide qualified lysin candidates for experimental verification.

### 2.3 Characterization of Putative Lysins and Identification of Potent LLysSA9

We assessed the accuracy of DeepLysin by experimentally verifying the antibacterial activity of the seventeen selected putative lysins against a diverse panel of bacterial species. These strains contained a variety of antibiotic‐resistance genes that provide resistance against antibiotics with diverse mechanisms of action (protein synthesis inhibitors, DNA/RNA synthesis inhibitors, cell wall synthesis inhibitors, and cell membrane disrupters) (Figure 4c). Five lysins could not be obtained even after three rounds of expression, while seven exhibited antibacterial activity via plaque formation, a success rate of 41.2% (7/17). Of the active lysins, LLysSA9 exhibited the broadest lysis activity, killing all tested strains of *Staphylococci* (*Staphylococcus aureus*, *Staphylococcus epidermidis*, *Staphylococcus haemolyticus*, *Staphylococcus capitis*, *Staphylococcus saprophyticus*, *Staphylococcus caprae*, *Staphylococcus pseudintermedius*, and *Staphylococcus hominis*) and *Streptococcus suis* (Figure 4c; these clinical strains each contained at least two antibiotic‐resistance genes), although activity against Gram‐negative ESKAPE pathogens (*Escherichia coli*, *Klebsiella pneumoniae*, *Acinetobacter baumannii*, *Pseudomonas aeruginosa*) was lacking (data not shown; the strains *tested are shown in Table* S5, Supporting Information). The activity observed with the putative lysins against problematic pathogens containing a variety of resistance genes demonstrates the ability of DeepLysin to provide potent candidates for use against multidrug‐resistant bacterial strains.

Given the excellent in vitro activity of LLySA8 and especially LLysSA9 against Gram‐positive pathogens in preliminary experiments, their antibacterial activity was further examined against 79 clinical isolates of methicillin‐susceptible *S. aureus* (MSSA) and 53 clinical isolates of methicillin‐resistant *S. aureus* (MRSA). These strains encompass the 57 major sequence types of clinical *S. aureus* isolates found globally (Figure S7, Supporting Information). The potent inhibitory activity of LLysSA9 (MIC_90_ of 4 µg mL^−1^ for MSSA and 2 µg mL^−1^ for MRSA) was substantially greater than that of CF‐301, the first lysin to enter phase III clinical trials (**Table** **1**; Table S6, Supporting Information). The activity of LLySA8 approximated that of CF‐301. As the CHAP domain of each lysin forms a groove that accommodates peptidoglycan, the lower binding energy between LLysSA9 and peptidoglycan may explain the greater activity observed (Figure S8, Supporting Information). These results show that our selected candidates, especially LLysSA9, exhibit potent activity against the major global sequence types of *S. aureus*, including multidrug‐resistant strains.

**Table 1:** MICs and MBCs (µg mL^−1^) of LLysSA9, LLysSA8, and CF‐301.

Lysin	Bacterium	*n*	MIC_50_	MIC_90_	Range	MBC_50_	MBC_90_	Range
LLysSA9	MSSA	79	2	4	0.125‐32	2	4	0.125‐32
	MRSA	53	1	2	0.125‐16	2	4	0.125‐32
LLysSA8	MSSA	79	16	32	4‐128	16	32	4‐128
	MRSA	53	16	32	4‐32	16	32	4‐32
CF‐301^a^ ^)^	MSSA	16	8	16	4‐32	8	16	4‐64
	MRSA	50	16	32	4‐128	16	32	4‐128
CF‐301^b^ ^)^	MSSA	74	16	32	8‐32	NA^c^ ^)^	NA^c^ ^)^	NA^c^ ^)^
	MRSA	75	32	32	2‐128	NA^c^ ^)^	NA^c^ ^)^	NA^c^ ^)^

^a)^ CF‐301 was expressed, purified, and tested under the same conditions as LLysSA8 and LLysSA9;

^b)^ Data collected from the literature;^[^ ^16^ ^]^

^c)^ No published data available.

### 2.4 Comparison of LLysSA9 with Traditional Antibiotics In Vitro

**Figure 5:**
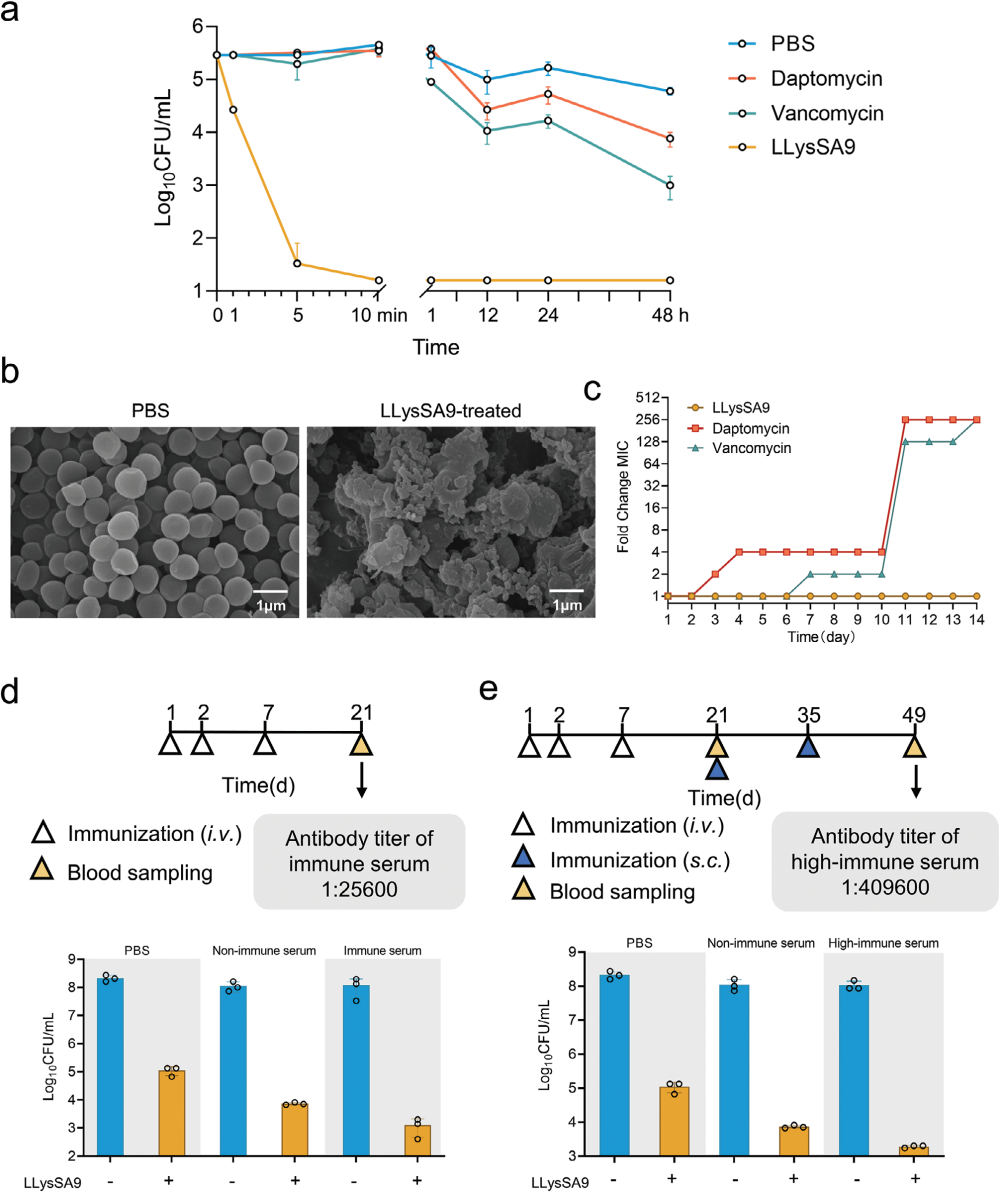
Antibacterial activity and immunological effects of LLysSA9. All experiments use *S. aureus* USA300. a) Time‐kill curves were generated for LLysSA9 (50 µg mL^−1^), vancomycin (50 µg mL^−1^), and daptomycin (50 µg mL^−1^) monotherapy against *S. aureus* in PBS, with a starting inoculum of ≈10^5^ CFU mL^−1^. Vancomycin and daptomycin acted as positive controls. b) SEM showing the effect on the bacterial membrane of 1 h exposure to 100 µg mL^−1^ LLysSA9, with a starting inoculum of ≈10^7^ CFU mL^−1^ (scale bar, 1 µm). c) Resistance‐acquisition in *S. aureus* following daily passaging for 14 d in the presence of LLysSA9, daptomycin, or vancomycin, at half the MIC. MICs were determined daily. d,e) Schematic diagrams showing the preparation of d) rat immune serum or e) rat high‐immune serum and the effect of immune serum/high‐immune serum on LLysSA9 bactericidal activity. Log‐phase *S. aureus* was exposed to 50 µg mL^−1^ LLysSA9 in PBS, non‐immune serum, and immune serum/high‐immune serum for 1 h.

Given the exceptional activity of LLysSA9 against clinical strains of *S. aureus*, further in vitro testing was undertaken. Time‐kill studies clearly showed rapid and potent bactericidal activity by LLysSA9 against *S. aureus* USA300, with no viable bacteria detected 10 min after treatment commenced and no regrowth up to 48 h. In stark contrast, reductions of only approximate 0.7 and 1.6 log_10_CFU mL^−1^ for daptomycin, and 1.2 and 2.5 log_10_CFU mL^−1^ for vancomycin, were observed at 24 and 48 h, respectively (**Figure** **5**a). Treatment with LLysSA9 caused significant membrane damage (Figure 5b) and the rapid release of bacterial contents (Video S1, Supporting Information). Furthermore, varying the temperature (4 °C, 25 °C, 37 °C), NaCl concentration (0–1000 × 10^−3^ m), urea concentration (0–500 × 10^−3^ m), or pH (5–12) had virtually no effect on activity (Figure S9, Supporting Information). Remarkably, the MIC of LLysSA9 did not change even after 14 d of daily passaging of *S. aureus* USA300 at half the MIC concentration, while the MICs of daptomycin and vancomycin increased 256‐fold (Figure 5c). The properties exhibited by LLysSA9 are highly desirable when treating serious infections associated with antibiotic‐resistant bacteria.

The production of antidrug antibodies (ADA) in response to lysin treatment is clinically important, given the potential interference with lysin catalytic activity.^[^ ^17^ ^]^ To address this concern, immune serum (1:25600) and high‐immune serum (1:409600) was used to test their effect on LLysSA9 activity. Surprisingly, both serums increased bacterial killing by approximately 1.5 log_10_ CFU/mL (Figure 5d,e). These preliminary results show LLysSA9 to be safe and effective following short‐term and long‐term multiple dose therapy, with antibodies not reducing (and possibly even enhancing) its activity.

### 2.5 LLysSA9 Successfully Combats Systemic Bacterial Infections In Vivo

**Figure 6:**
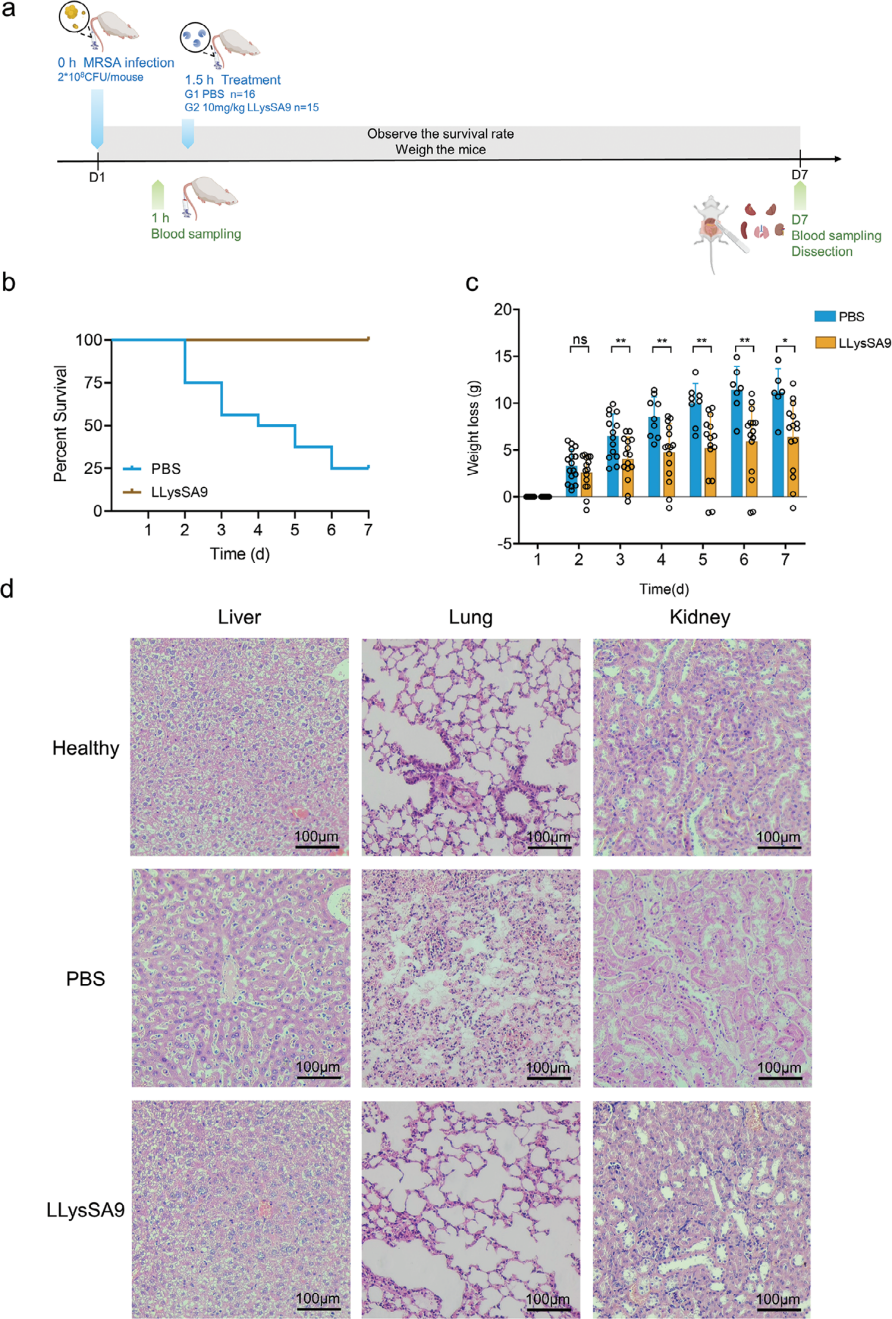
Mouse bacteremia model. a) Experimental setup of the mouse bacteremia model. Mice were intravenously (i.v.) injected with MRSA USA300 (2 × 10^8^ CFU per mouse). After 1.5 h of infection, i.v. administration of PBS (control group) or 10 mg kg^−1^ LLysSA9 was performed. Immediately prior to treatment and after 7 d, blood was collected for bacterial counting. Dead mice were promptly dissected, and their organs (heart, liver, spleen, lungs, and kidneys) collected for bacterial counting. On day 7, surviving mice were euthanized and their organs dissected for bacterial counting. b) Survival curves, and c) changes in body weight for control and LLysSA9‐treated mice. d) Histological evaluation of representative organs (liver, lung, kidney) stained with H&E in healthy mice, infected mice (controls), and LLysSA9‐treated mice. Scale bar, 100 µm.

**Figure 7:**
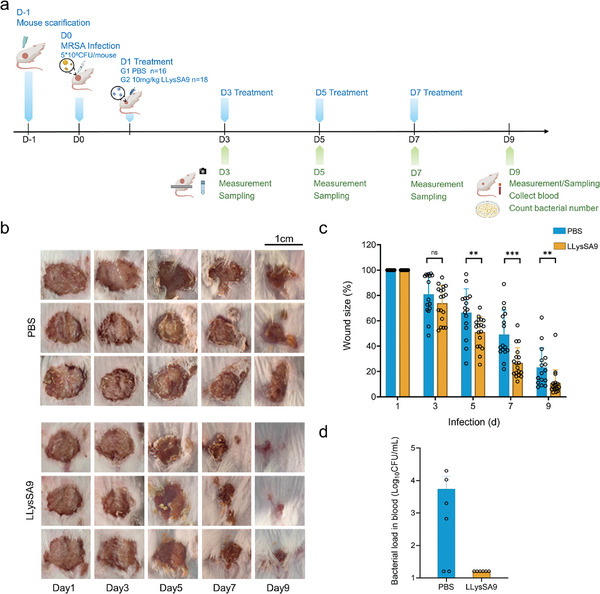
Systemic infection from mouse skin wound infection model. a) Experimental setup of the mouse skin wound infection model. Skin infection was established via subcutaneously (s.c.) injection of MRSA USA300 (5×10^8^ CFU per mouse). PBS (controls) or LLysSA9 (10 mg kg^−1^) was dropped on the wounds. On days 1, 3, 5, and 7, treatment was administered, the wound photographed, and bacteria sampled from the wound for viable counting. After 9 d, blood was collected for viable counting and the mice euthanized. b) Representative photographs of wounds from the control and treatment groups (n = 3 mice per group). c) The wound size (%) of mice from each group at days 1, 3, 5, 7, and 9 are shown. The data were pooled from two independent experiments (*n* = 16 mice in PBS group; *n* = 18 mice in LLysSA9 group). d) Bacterial load in blood after 9 d of treatment (*n* = 6 mice per group). The *y* axis starts at the limit of detection.

The in vivo effectiveness of LLysSA9 against *S. aureus* was examined in mouse bacteremia (**Figure** **6**a) and skin infection (**Figure** **7**a) models using *S. aureus* USA300 to assess intravenous and topical use, respectively. Cytotoxicity assays performed prior to in vivo testing showed LLysSA9 at concentrations up to 100 µg mL^−1^ (equivalent to 100 times the MIC_50_) had negligible cell toxicity (Figure S10, Supporting Information).

In the bacteremia model, at day 7 LLysSA9 significantly reduced mortality (from 75% in control mice versus no deaths in LLysSA9‐treated mice; Figure 6b), bacterial loads in the blood and organs (Figure S11a, Supporting Information), and weight loss (Figure 6c). During the 7‐day period, control mice exhibited various clinical signs following bacterial challenge that were not observed in LLysSA9‐treated mice, including decreased locomotor activity, piloerection, and flocking behavior (Figure S11b, Supporting Information). Severe abscesses present in the kidneys of control mice were dramatically attenuated with LLysSA9 treatment (Figure S11c, Supporting Information). Tissue histology showed pathological changes in the main organs of control mice including interstitial hyperplasia and obvious inflammatory cell infiltration of the lung, nuclear dissolution of the kidney, and ground‐glass hepatocyte; LLysSA9‐treated mice showed a relatively normal histological results consistent with healthy mice (Figure 6d). These findings underscore the significant potential of LLysSA9 to treat systemic MRSA infections.

Finally, in the skin wound infection model, topical LLysSA9 increased the rate of wound healing, reducing the size of the wounds on day 9 by 19.4% compared to control mice (Figure 7b; Figure S12, Supporting Information). Importantly, LLysSA9 treatment not only reduced the bacterial load in the wound compared to control mice (Figure 7c), but also eliminated bacteria from the blood (Figure 7d).

## 3 Discussion

In a world where the once‐reliable arsenal of antibiotics is losing its effectiveness against bacterial infections, and the discovery of new antibiotics is dwindling, we are on the brink of a crisis reminiscent of the pre‐antibiotic era.^[^ ^18^ ^]^ To prevent such a catastrophic regression, we urgently need new platforms for discovering antibacterial drugs to replenish our diminishing stocks of effective antimicrobials. Lysins are promising candidates against bacterial infections. These enzymes have demonstrated efficacy against both Gram‐positive and Gram‐negative pathogens, with each of the problematic drug‐resistant ESKAPE pathogens having corresponding lysins.^[^ ^19^ ^]^ Of particular appeal is their rapid bactericidal activity and limited potential for the development of bacterial resistance, traits that are invaluable in the battle against multidrug‐resistant bacteria. However, despite their potential, the development of lysins from discovery to clinical use has been slow. Since their initial identification in 1971,^[^ ^20^ ^]^ less than 2% of lysins have been identified and validated experimentally, with only five reaching the clinical trial stage. Clearly, there is a pressing need for more effective methods to identify potential therapeutic lysins.

Here, we propose the first conceptual application that utilizes artificial intelligence to achieve global exploration and activity evaluation of antibacterial proteins from the vast amounts of available genetic “dark matter.” The development of multidimensional data analysis techniques, such as modern statistical graphics and artificial intelligence, has forged a direct link between biomedical and computational science. Yet, as we stand on the cusp of a new era where the wealth of metagenomic and genomic data is multiplying at an unprecedented rate, fully harnessing the potential of these advancements for drug discovery presents a formidable challenge. To navigate this terrain effectively, we require sophisticated techniques capable of accurately identifying potential clinical protein candidates and meticulously quantifying their antimicrobial activity during the data excavation process.^[^ ^21^ ^]^ This dynamic landscape of continuously expanding genome reservoirs and the increasingly important role of artificial intelligence inspired us to confront these two critical challenges head‐on.

While existing resources like the cloud‐based database PhaLP^[^ ^22^ ^]^ were designed to compile known lysins from UniProt, they fail to provide the systematic and high‐throughput support needed to process and mine the massive amounts of sequencing data essential for concise screening of novel lysins. Similarly, while platforms like the VersaTile‐driven system excel in constructing combinatorial libraries of engineered lysins and conducting iterative screening of active lysins, the reliance of each of their modules (peptide, linker, EAD, and CBD) on reported sequences limits their ability to uncover truly novel sequences.^[^ ^10a^ ^]^ Other methods that rely on building combinatorial libraries also face the same issue.^[^ ^23^ ^]^ Prior to our development of DeepLysin, no software or pipeline was available to fully utilize sequencing resources to conduct de novo screening of novel lysins and evaluate their activity. The *DeepLysin software* is a comprehensive and intuitive tool that can be adapted to explore all available lysins for activity against diverse pathogens. *The two modules that comprise* DeepLysin *form a unified software package for lysin identification and activity evaluation* from the vast expanse of available “dark matter” data sources. Its step‐by‐step lysin mining module greatly simplifies the challenge of mining large‐scale datasets, enabling rapid screening of drug candidates for activity against virtually any bacterium from assembled genomes/metagenomes in a fraction of the time previously required (typically about 1 hour in the phage library and 3 d in the bacteria library; Tables S7 and S8_,_ Supporting Information). Furthermore, DeepLysin can also be used for prophage prediction or mining antibacterial proteins which can interact with peptidoglycan.

During development of the lysin mining module, the least likely lysin candidates were initially filtered. Artificial intelligence was then introduced to further exclude putative lysins without predicted activity. It became apparent that relying solely on individual machine learning models like GLM or SVM was insufficient to discern between active and inactive lysins. In designing the first layer of stacking model, we considered a combination of complementary models to utilize the advantages of each model to better identify small differences in the high‐dimensional features of active proteins (AAC, BPNC, GAAC, CTD, AAE, and AAI; Figure 2e), thereby efficiently discarding putative lysins devoid of predicted activity. This process provided a crucial insight into how artificial intelligence perceives the distinctive features of antibacterial proteins: the amalgamation of five machine learning models, each contributing its unique perspective, precisely extracted the subtle nuances in the high‐dimensional features of antibacterial proteins. We leveraged this newfound understanding to develop the stacking model to identify lysins predicted to have antibacterial activity.

We computationally screened a library of more than 4000 putative lysins from uncharacterized *S. aureus* phages. The putative lysins covered all reported experimentally verified lysins (Table S2, Supporting Information), which are considered as positive results, confirming the effectiveness of DeepLysin. We then excluded sequences with high similarity to reported lysins and searched for new lysin sequences in the remaining library. By excluding sequences with high similarity to reported lysins (thereby narrowing down the positive results) while maintaining a stable number of negative results in the putative lysin library, we established a more rigorous method to evaluate DeepLysin's ability to identify novel lysins. Using these criteria, we randomly selected 17 candidates for synthesis and experimental validation. Seven lysins (41.2%) exhibited excellent antibacterial activity against the tested Gram‐positive pathogens, each having a distinct spectrum of activity. Importantly, DeepLysin was able to identify lysins with novel unreported domain architecture, thereby providing greater structural diversity for the design of new therapeutic lysins.^[^ ^24^ ^]^ The continued expansion of experimentally verified lysins will likewise expand the pool of positive and negative samples available for model training, enabling further optimization of the DeepLysin software to enhance accuracy. Further improvements could be made by incorporating structural information critical for lysin function using in‐depth profiling of protein structure and ligand interactions, thereby determining the relationship between structure and activity. This would provide more dimensional data support for lysin mining.

Bacteremia caused by *S. aureus* is a significant problem globally and has the highest risk of mortality compared to other bacteremia‐causing pathogens (as high as 30% at 30 d).^[^ ^25^ ^]^ Treating such infections is becoming increasingly difficult due to the emergence of drug‐resistant strains.^[^ ^26^ ^]^ Acknowledging the substantial health threat posed by *S. aureus*, we elected to evaluate LLysSA9, the most promising putative lysin identified by DeepLysin, both in vitro against multiple MRSA and non‐MRSA clinical strains, and in vivo against a single clinical MRSA strain. LLysSA9 and comparator lysin CF‐301, the first lysin to enter a phase III trial, were tested by broth microdilution against a set of clinical MSSA and MRSA isolates. The MIC_50_ of LLysSA9 was 4–16 times lower than that of CF‐301, with greater antibacterial activity. LLysSA9 eliminated bacteria within 10 min of the commencement of therapy in time‐kill studies, with no resistance development after 14 d of daily passaging. Its effectiveness was also clearly observed in the bacteremia and wound infection models. Thus, we have provided the first systematic proof that best‐in‐class candidate can be mined from “dark matter.” These promising findings underscore the significant potential of DeepLysin to identify therapeutically active lysins from “dark matter,” thus greatly assisting efforts to counteract the escalating global antibiotic resistance crisis.

## 4 Conclusion

In conclusion, we describe here the development of DeepLysin, the first conceptual application to employ artificial intelligence in the discovery of antibacterial proteins, demonstrating its ability to achieve high‐throughput screening of lysins from “dark matter.” We also report how artificial intelligence identifies the features necessary for active protein candidates. One therapeutic lysin candidate identified by DeepLysin, LLysSA9, displayed excellent in vitro activity and therapeutic efficacy in mouse bacteremia and skin wound infection models. DeepLysin is thus able to generate promising antibacterial protein drug candidates to combat clinically relevant bacteria, including problematic multidrug‐resistant strains.

## 5 Experimental Section

### Collections of Typical Lysin EADs and CBDs

PubMed (April 17, 2023) was utilized to identify relevant studies in the English language using the keywords “lysin” and “*Staphylococcus*.” A total of 636 publications describing 67 lysins active against *S. aureus* and with publicly available sequences were identified. In addition, 41 typical EADs, 23 typical CBDs, and 23 unknown domains from any reported lysins were summarized for subsequent lysin mining (Table S9, Supporting Information).

### Dataset Preparation

The 148269 positive lysin sequences collected from NCBI (https://www.ncbi.nlm.nih.gov/) comprised patents and experimentally validated lysin sequences. The negative dataset was generated as described previously.^[^ ^27^ ^]^ Protein sequences with the highest annotation level and containing 100–400 amino acids were downloaded from Uniprot.^[^ ^28^ ^]^ The negative dataset contained 3863 lysin sequences, excluding those annotated as “antimicrobial, hydrolase, hemolysin, lysin, glycosidase, endolysin, holin, and bactericidal.” After removing redundancy by CD‐hit using parameters “‐c 0.9,” 16135 positive sequences and 2952 negative sequences remained. Sequences containing unnatural amino acids (B, J, O, U, X, and Z) were then removed, leaving 16087 positive sequences and 2921 negative sequences. To ensure an appropriate balance between positive and negative samples, sequences from the positive samples were randomly sampled (a number approximately equal to the negative samples) for subsequent training. These samples were split into the training and testing datasets at a ratio of 7:3. The training dataset was used to build the stacking model and the testing dataset to evaluate the performance of the stacking model.

### Testing Data Preparation

The genomes of *S. aureus* were downloaded from NCBI (January 26, 2023) and those of *S. aureus* phages from MVP, PhagesDB, and VHDB.^[^ ^29^ ^]^ CheckM^[^ ^30^ ^]^ and CheckV^[^ ^31^ ^]^ were used to evaluate genome integrity and exclude data with an integrity of <90%.

### Putative Lysin Mining

The putative lysin mining method was supported in metagenomes and single‐genomes data. First, the “‐bp” parameter was set for selecting different types of genomes (bacteria or phage). For the bacterial genomes/metagenomes, CDS was predicted using prokka 1.14.6,^[^ ^32^ ^]^ prophages annotated using Phispy 4.2.21,^[^ ^33^ ^]^ and the coordinate of prophages extracted using DeepLysin. The functional protein sequences from prophages and phages were annotated by prokka. Second, CD‐hit^[^ ^34^ ^]^ was used to remove protein sequence redundancy; if necessary, additional protein segments were removed based on user‐defined molecular mass using ‐mL and ‐mu. Third, “HMMsearch”^[^ ^35^ ^]^ was employed to match protein sequences containing typical domains, with an EAD as a requirement. Finally, protein sequences containing transmembrane domains, predicted by DeepTMHMM 2.0,^[^ ^36^ ^]^ were customarily removed.

### Features Extraction

Six features (AAC, BPNC, AAE, AAI, GAAC, CTD) divided into four categories were provided to capture protein sequence characteristics.^[^ ^37^ ^]^ The detailed information of features is listed in Table S10, Supporting Information.

### Stacking Model Training

As a representative two‐layer learning method, the stacking model framework trained five machine learning classifiers (ANN, ERT, KNN, LR, and XGBoost) in the first layer using different combinations of features (AAC, BPNC, AAE, AAI, GAAC, CTD). Silhouette Coefficient was performed to evaluate the clustering effect of eigenvectors extracted in the first layer^[^ ^38^ ^]^ and calculated as below:

(1) $$s\left(i\right)\;=\frac{b\left(i\right)-a\left(i\right)}{\max\left[a\left(i\right),\;b\left(i\right)\right]}\;$$

where *s*(i) is the Silhouette coefficient, *a*(i) is the distance from point i to all the other points in the same cluster, and *b*(i) is the average distance from point i to all points in other clusters which do not contain point i. The R package “Rtsne” was then used for dimensionality reduction visualization of eigenvectors.^[^ ^39^ ^]^ The second layer was then trained using LR classifier based on the outputs of the five basal models of the first layer. The output of the second layer was the final result and ranged between 0 and 1. The user‐friendly model training method for selecting different combinations of models was provided in the custom script “Train_costom.py.”

### Performance Evaluation of Models

The performance of models was evaluated by accuracy (ACC), Matthew's correlation coefficient (MCC), F1 score, the area under curve (AUC), and the area under the precision–recall curve (AUPRC), all commonly used in binary classification.^[^ ^40^ ^]^ They were calculated as below:

(2) $$\mathrm{ACC}\;=\frac{\mathrm{TP}+\mathrm{TN}}{\mathrm{TP}+\mathrm{TN}+\mathrm{FP}+\mathrm{FN}}\;$$

(3) $$\mathrm{MCC}\;=\frac{\mathrm{TP}\times \mathrm{TN}+\mathrm{FP}\times \mathrm{FN}}{\sqrt{\left(\mathrm{TP}+\mathrm{FN}\right)\left(\mathrm{TP}+\mathrm{FP}\right)\left(\mathrm{TN}+\mathrm{FP}\right)\left(\mathrm{TN}+\mathrm{FN}\right)}}\;$$

(4) $$\mathrm{Recall}\;=\frac{\mathrm{TP}}{\mathrm{TP}+\mathrm{FN}}\;$$

(5) $$F1\;\mathrm{score}\;=\frac{2\mathrm{TP}}{2\mathrm{TP}+\mathrm{TP}+\mathrm{FN}}\;$$

where TP, TN, FP, and FN indicate the number of true positive, true negative, false positive, and false negative samples, respectively.

### Phylogenetic Analysis

Multiple sequence alignment of putative lysins and reported lysins was performed using Clustal Omega (https://www.ebi.ac.uk/Tools/msa/clustalo/). A phylogenetic tree was built by maximum likelihood with 500‐bootstrap replication using MEGA (version 11) and visualized using iTOL (https://itol.embl.de/).

### Network Analysis

The EAD and CBD of putative and reported lysins were split by the prediction results of sequences in the CD search database (https://www.ncbi.nlm.nih.gov/Structure/bwrpsb/bwrpsb.cgi). CD‐hit was used to cluster the amino acid sequences of the domains, with a threshold of similarity >80%. To build the network, the EAD and CBD domains were the nodes, and co‐occurrences of EAD and CBD in the same lysin were linked. The network‐based relationships between domains were visualized using Gephi (version 10.1).

### 3D Structure Analysis and Molecular Docking

The 3D structure of putative lysins was predicted by AlphaFold2.^[^ ^41^ ^]^ Available peptidoglycan structures were downloaded from the PDB database with some revisions (https://www.rcsb.org/). AutoDock (v4.2.6) was used to perform docking simulations between putative lysins and peptidoglycan fragments. The Genetic Algorithm was run 100 times to screen the optimal protein‐ligand compound and ensure accurate molecular docking results. The compound was analyzed by Pymol (v2.5.5) and interactions on a 2D diagram visualized by Discovery Studio (version 2019).

### Bacterial Strains and Cell Growth Conditions

All bacteria used are listed in Tables S5 and S6 (Supporting Information). Streptococcus strains were cultured aerobically in tryptic soy broth or agar containing 5% newborn calf serum, and other strains in Luria Bertani broth (LB) or agar (LA). All strain were incubated overnight at 37 °C with shaking at 200 rpm. Log‐phase cultures were prepared by incubating a 1 in 50 dilution of overnight culture for 2–4 h at 37 °C until an absorbance of 0.5–0.6 at 600 nm was reached. Cells were cultured in Dulbecco's modified eagle medium: nutrient mixture F‐12 supplemented with 10% fetal bovine serum at 37 °C in a humidified atmosphere containing 5% CO_2_.

### Cloning, Expression, and Purification of Lysins

Genes of putative lysins were commercially synthesized into plasmid pET14b (Sangon Biotech, Shanghai). The resulting plasmids were transformed into E. coli BL21(AI) using a CaCl_2_ procedure. Log‐phase BL21(AI) was induced with 1 × 10^−3^ m IPTG (isopropyl‐β‐D‐thiogalactopyranoside) and 0.15% L‐arabinose (w/v) at 18 °C for 20 h to allow protein expression. Bacteria were then centrifuged (4000 rpm for 20 min), resuspended in the binding buffer (50 × 10^−3^ m Tris pH 8.0, 0.5 m NaCl, 10% glycerol), disrupted by homogenization, and centrifuged (10 000 rpm for 30 min at 4 °C) to remove cell debris. Lysates were loaded on a nickel‐nitrilotriacetic acid (Ni‐NTA) column (HisTrap HP, Sangon Biotech) and the proteins were purified using an AKTA Pure Protein Purification System (GE Healthcare Life Sciences). The eluted fractions were dialyzed against HEPES buffer (30 × 10^−3^ m, pH 7.4) in a 14‐kDa‐cutoff membrane. Proteins were concentrated using an Amicon ultrafiltration device with a 10‐kDa‐cutoff membrane, and a bicinchoninic acid assay (Biosharp) was used to determine the final concentration. The purity of proteins was confirmed by SDS‐PAGE.

### Preliminary Screening of Lysin Activity

All in vitro experiments were performed three times. Lysate (5 µL) was spotted onto double‐layer agar containing bacterial lawn. The tested bacterial strains included *Staphylococci*, *Streptococci*, and other Gram‐negative strains (*Escherichia coli*, *Klebsiella pneumoniae*, *Acinetobacter baumannii*, and *Pseudomonas aeruginosa*) (Table S5, Supporting Information). Lysins were deemed to have bactericidal activity if plaque formation was observed.

### MIC Determination

The minimum inhibitory concentrations (MICs) of LLysSA8, LLysSA9, and CF‐301 were determined in 96‐well plates using broth microdilution as per the Clinical and Laboratory Standards Institute (CLSI) guidelines.^[^ ^42^ ^]^ Briefly, *Staphylococci* strains (5 × 10^5^ CFU mL^−1^) were incubated with different concentrations of lysin (0.25–256 µg mL^−1^) in cation‐adjusted (0.45 × 10^−3^ m) Mueller Hinton broth (CaMHB) at 37 °C for 18 h. The MIC was defined as the lowest concentration of lysin without visible bacterial growth. The reaction solution from each well was spotted onto LA and the minimum bactericidal concentration (MBC) was determined. The MBC was defined as the minimum concentration of lysin without colony growth.

### Killing Kinetics

For investigation of time‐kill kinetics, suspensions of *S. aureus* USA300 in PBS (≈10^5^ CFU mL^−1^, 150 µL) were treated with an equal volume of PBS (10 ×10^−3^ m, pH 7.4; control groups), LLysSA9 (50 µg mL^−1^), or antibiotic (datomycin or vancomycin; 50 µg mL^−1^), and incubated at 37 °C with shaking (200 rpm) for 48 h. Samples (25 µL) for viable counts were collected aseptically at 0, 1, 5, and 10 min, and 1, 12, 24, and 48 h, serially diluted with PBS, and plated on LA for enumeration. The bactericidal activity of LLysSA9 under different biochemical conditions is described in the Supporting Information.

### Drug Resistance

Drug resistance of *S. aureus* was tested by daily passaging for 14 d with LLysSA9, vancomycin, or daptomycin at half the MIC. Bacterial MICs were determined by broth microdilution after every passage. Between every passage, the bacteria (100 µL) in the well at half the MIC were transferred to CaMHB (5 mL) and cultured to log‐phase (37 °C, 2–3 h), which served as inoculum for the next passage. Drug resistance was considered to have emerged if the initial MIC increased fourfold.

### Scanning Electron Microscopy (SEM)

Suspensions of *S. aureus* (10^7^ CFU mL^−1^) were treated with either PBS (10 × 10^−3^ m, pH 7.4; control groups) or LLysSA9 (100 µg mL^−1^) for 1 h. Bacterial lysates were harvested, fixed with 2.5% glutaraldehyde at 4 °C for 4 h, and dehydrated in gradient ethanol. Following precooling at −80 °C for 4 h, dried samples were attached to an aluminum conductive adhesive and sprayed with gold using an ion sputtering apparatus. All images were captured using a scanning electron microscope (JSM‐6390LV). Inverted phase contrast microscopy was also used (described in the Supporting Information).

### Preparation of Immune Serum and Its Effect on Lysin Bactericidal Activity

Cytotoxicity assays performed prior to in vivo testing are described in the Supporting Information. All animal experiments were performed in accordance with the operational procedures of the Experimental Animal Center of Huazhong Agricultural University. A single intravenous (i.v.) injection of LLysSA9 (10 mg kg^−1^, 1 mL) or PBS (1 mL; controls) was administered to Sprague‐Dawley (SD) rats (13 weeks old, female; 3 rats per group) on days 1, 2, and 7. Fourteen days after the last injection, blood samples (1 mL) were collected via the tail vein, allowed to stand at room temperature for 30 min, and centrifuged (4000 rpm, 10 min) to obtain immune serum. The LLysSA9 antibody titer was checked by enzyme‐linked immunosorbent assay (ELISA). A neutralization assay was performed by mixing LLysSA9 (final concentration 50 µg mL^−1^) with LLysSA9 immunized or nonimmunized rat serum, followed by incubation at 37 °C for 1 h. An aliquot (50 µL) of the mixture was then added to an equal volume of *S. aureus* USA300 (suspended in PBS), incubated at 37 °C for 1 h, serially diluted with PBS, and plated on LA for viable counting.

High‐immune serum was prepared using the above method with the addition of two subcutaneous injections (4 mg kg^−1^; same volume as per IV), one 14 d and one 28 d after the third i.v. injection. Blood samples were collected 14 d after the second subcutaneous injection. Then, ELISA and neutralization assay were performed. Experiments were performed in duplicate (6 rats per group in total).

### Efficacy of LLysSA9 in Bacteremia Mouse Models

In the bacteremia model, 31 female Kunming (KM) mice (5 weeks old) were intravenously injected with *S. aureus* USA300 (2 × 10^8^ CFU per mouse). After 1 h of bacterial infection, blood samples (200 µL; 16 mice) were collected through the tail vein for colony counting. At 1.5 h, mice were randomly divided into two groups that received a single intravenous dose of either LLysSA9 (10 mg kg^−1^; 15 mice) or PBS buffer (16 mice). Experiments were conducted across 7 d. Clinical symptoms were observed, and body weights and the number of dead mice were recorded daily. The dead mice were promptly dissected, and their organs (heart, liver, spleen, lungs, and kidneys) were collected. After 7 d, blood samples were collected, mice were euthanized by inhalation of isoflurane, and major tissues were collected. Pathological changes were photographed and examined following immersion in 4% paraformaldehyde and H&E staining. Bacterial enumeration of blood and organs was also performed. This experiment was performed in duplicate, with the 31 mice covering both experiments.

### Efficacy of LLysSA9 in Skin Wound Infection Mouse Models

The skin wound infection mouse model was established on the BALB/c mice (9 weeks old, female; 6 mice per group). Another repeated experiment was established on the KM mice (9 weeks old, female; 10 mice in PBS group, 12 mice in LLysSA9 group). After shaving the dorsal fur, each mouse received a 1 cm diameter round full‐thickness wound induced by punch, with *S. aureus* USA300 (5 × 10^8^ CFU per mouse) then injected subcutaneously around the wound. After 24 h, a subcutaneous abscess formed at the infection site, indicating the successful establishment of infection. Infected mice were randomly divided into two groups that received treatment on days 1, 3, 5, and 7 with either PBS (control group) or LLysSA9 (10 mg kg^−1^), administered by dropping (via a pipette) onto the wound. On days 3, 5, 7, and 9, each wound was photographed including a ruler for measurement and bacterial samples were taken using a cotton swab for viable counting. After 9 d, blood was collected for viable counting and the mice were euthanized. The wound size was calculated by Adobe Illustrator 2020. This experiment was performed in duplicate.

### Statistical Analyses

All statistical analysis was performed using GraphPad Prism 9.0. Data are presented as mean ± standard deviation. The statistical significance of different groups was based on Student's t test or a Mann‐Whitney U test (*p < 0.05; **p < 0.01; ***p < 0.001; ns, not significant).

## Conflict of Interest

The authors declare no conflict of interest.

## Supporting information

Supporting Information

Supplemental Video 1

## Data Availability

The data that support the findings of this study are available from the corresponding author upon reasonable request.
