# Supplementary material for: Discovery of Antimicrobial Lysins from the “Dark Matter” of Uncharacterized Phages Using Artificial Intelligence
Source: Adv Sci (Weinh). 2024 Jun 20;11(32):2404049. doi: 10.1002/advs.202404049 (PMC11348152; doi:10.1002/advs.202404049)
Supplement: Supplementary file 1 — Supporting Information [file ADVS-11-2404049-s001.docx]

**Supplementary Information**

**Discovery of Antimicrobial Lysins from the ‘Dark Matter’ of Uncharacterized Phages using Artificial Intelligence**

*Yue Zhang^1^, Runze Li^1^, Geng Zou, Yating Guo, Renwei Wu, Yang Zhou, Huanchun Chen, Rui Zhou, Rob Lavigne, Phillip J. Bergen, Jian Li, and Jinquan Li^*^*

Y. Zhang, R. Li, G. Zou, Y. Guo, R. Wu, Y. Zhou, H. Chen, R. Zhou, J. Li*^*^*

National Key Laboratory of Agricultural Microbiology, Key Laboratory of Environment Correlative Dietology, College of Biomedicine and Health, Shenzhen Institute of Nutrition and Health, Huazhong Agricultural University, Wuhan 430070, China

Y. Zhang, R. Li, G. Zou, J. Li*^*^*

Hubei Hongshan Laboratory, College of Food Science and Technology, Huazhong Agricultural University, Wuhan 430070, China

Y. Guo, R. Wu, H. Chen, R. Zhou, J. Li*^*^*

College of Veterinary Medicine, Huazhong Agricultural University, Wuhan 430070, China

J. Li*^*^*

Shenzhen Branch, Guangdong Laboratory for Lingnan Modern Agriculture, Genome Analysis Laboratory of the Ministry of Agriculture and Rural Affairs, Agricultural Genomics Institute at Shenzhen, Chinese Academy of Agricultural Sciences, Shenzhen 518000, China

R. Lavigne

Department of Biosystems, Laboratory of Gene Technology, KU Leuven, Leuven 3001, Belgium

P. J. Bergen, J. Li

Monash Biomedicine Discovery Institute, Department of Microbiology, Faculty of Medicine, Nursing and Health Sciences, Monash University, Melbourne 3800, Australia

^1^These authors contributed equally to this work.

***Corresponding author**

J. Li, E-mail: [lijinquan2007@gmail.com](mailto:lijinquan2007@gmail.com).

**Keywords:** phage lysin, high-throughput screening, stacking model, antibacterial protein, antibiotic resistance, infectious diseases, prophage

**Supplementary Methods**

**The bactericidal activity of LLysSA9 under different conditions**

Log phase bacteria were diluted to 10^5^ CFU/mL in different buffers, including those with different pH values (30 mM acetate buffer [pH 5.0], MES [morpholineethanesulfonic acid] buffer [pH 6.0], HEPES buffer [pH 7.0 and 8.0], CHES [N-Cyclohexyltaurine] buffer [pH 9.0 and 10.0], CAPS [N-cyclohexyl-3-aminopropanesulfonic acid] buffer [pH 11.0], and Na_2_HPO_4_ [dibasic sodium phosphate] buffer [pH 12.0]), NaCl (0 to 1000 mM in 30 mM HEPES buffer), and urea (0 to 500 mM in 30 mM HEPES buffer). The bacteria were then incubated with 50 μg/mL LLysSA9 for 1 h at 37 °C with shaking (200 rpm), serially diluted, and plated on Luria Bertani agar for enumeration.

**Inverted phase contrast microscopy**

Poly-L-lysine (0.1 mg/mL) was coated on rectangular coverslips (24×50 mm) overnight at 4 °C. Subsequently, the bacterial solution (10^7^ CFU/mL) was spread onto the coverslips at room temperature for 10 min and the unattached bacteria removed with PBS. PBS containing 50% glycerol and 0.1% p-Phenylenediamine (pH 8.0) was dropped onto the coverslips and covered with a square coverslip (24×24 mm). The sample was placed on the stage of the inverted phase contrast microscope (Nikon Ti, Japan), and a suitable field of view found. Finally, lysin was added from one side of the coverslip, with absorbent paper used on the other side to capture the process of bacterial lysis.

**Cell cytotoxicity**

The biocompatibility of lysin with a variety of concentrations was investigated by MTT assay. RAW264.7 cells were seeded into each well of a 96-well plate and cultured overnight to 80% coverage. The culture medium was removed and different concentrations of LLysSA9 (6.25-100 μg/mL) in the medium added to each well. Cells were cultured for 24 h, the medium replaced by serum-free DMEM medium containing 0.5mg/mL 3-(4,5)-dimethylthiahiazo (-z-y1)-3,5-di- phenytetrazoliumromide (MTT), and incubated for another 4 h. The supernatant was then removed, DMSO added to each well to dissolve any crystals formed, and the mixture incubated at 37 °C with shaking (200 rpm) for 10 min. A570 was then measured. The wells without added LLysSA9 were set as 100% cell viability. Cell viability was calculated as:

$$\text{Cell viability}\left( \text{\%} \right)\text{=}\frac{\text{A}_{\text{sample}}}{\text{A}_{\text{blank}}}\text{×100}$$

**Supplementary Tables**

**Table S1.** The performance of the stacking model compared with other models.

| **Model** | **AUC** | **AUPRC** |
| --- | --- | --- |
| SVM (Two features) | 0.9152 | 0.6915 |
| SVM (Three features) | 0.9016 | 0.6972 |
| SVM (Four features) | 0.8828 | 0.6935 |
| SVM (Five features) | 0.8655 | 0.6939 |
| SVM (Six features) | 0.7257 | 0.6756 |
| GLM (Two features) | 0.9183 | 0.9190 |
| GLM (Three features) | 0.9208 | 0.9187 |
| GLM (Four features) | 0.9035 | 0.8999 |
| GLM (Five features) | 0.8846 | 0.8803 |
| GLM (Six features) | 0.9545 | 0.9570 |
| Stacking model | 0.9995 | 0.9995 |

**Table S2.** The blastp results between 67 reported lysins and 357 lysin candidates. The sequences of 67 reported lysins were set as ‘Query Sequence’, while the sequences of 357 lysin candidates were set as ‘Subject Sequence’. The following table only lists the most matching sequence of 67 reported lysins among 357 lysin candidates.

|  | **The information of reported lysins** | | | **The most matching sequence of 67 reported lysins among 357 candidates** | | | | |
| --- | --- | --- | --- | --- | --- | --- | --- | --- |
| **NO** | **Reported lysin** | **Natural/**  **Chimeric lysin** | **Origin / Construction** | **Lysin candidate** | **Query Cover** | **E value** | **Per. Ident** | **Acc. Len** |
| 1 | Abtn-4 | Natural lysin | Acinetobacter baumannii phage vB_AbaP_D2 | No significant similarity found. | | | | |
| 2 | CF-301 | Natural lysin | Streptococcus suis strain 89/1591 | LOKADMLJ_00065 | 32% | 7.00E-08 | 39.02% | 341 |
| 3 | ClyC | Chimeric lysin | Ply187 CHAP + LysSA97 non-SH3b | JPGJNENK_00022 | 61% | 2.00E-115 | 100.00% | 628 |
| 4 | ClyF | Chimeric lysin | Ply187 CHAP + CF-301 SH3b | JPGJNENK_00022 | 65% | 9.00E-116 | 95% | 628 |
| 5 | ClyH | Chimeric lysin | Ply187 CHAP + phiNM3 non-SH3b | JPGJNENK_00022 | 81% | 3.00E-115 | 79.44% | 628 |
| 6 | ClyL | Chimeric lysin | LysGH15 CHAP + Lys0859 SH3b | EAONIOPL_00076 | 74% | 2.00E-125 | 85.50% | 267 |
| 7 | ClyR | Chimeric lysin | PlyC CHAP + CF-301 SH3b | LOKADMLJ_00065 | 47% | 3.00E-08 | 33.09% | 341 |
| 8 | E-LM12 | Natural lysin | Staphylococcus aureus phage vB_SauM-LM12 | AIKLEEOI_00058 | 100% | 0 | 97.78% | 496 |
| 9 | Endo88 | Natural lysin | Staphylococcus aureus phage 88 | EBKFGABG_00027 | 100% | 0 | 100.00% | 481 |
| 10 | EnpACD | Natural lysin | Enterococcus faecalis prophage | GCF_003609895_1_pp1:R:2707-8907 | 80% | 1.00E-16 | 36.89% | 2066 |
| 11 | HydH5 | Natural lysin | Staphylococcus aureus phage PhiH5 | HDKPDOPD_00058 | 100% | 0 | 100.00% | 634 |
| 12 | Jdlys | Natural lysin | Staphylococcus aureus phage JD007 | GNAFGFLF_00055 | 100% | 0 | 100.00% | 495 |
| 13 | Ly7917 | Natural lysin | Streptococcus suis prophage phi7917 | KHMCFBFL_00024 | 32% | 8.00E-06 | 32.10% | 484 |
| 14 | LyJH1892 | Natural lysin | Staphylococcus aureus strain | NJCEENLM_00025 | 100% | 0 | 100.00% | 484 |
| 15 | Lys210 | Natural lysin | Staphylococcus aureus Jumbo phage vB_StaM_SA1 | DDLECFAO_00013 | 100% | 2.00E-48 | 34.04% | 249 |
| 16 | Lys211 | Natural lysin | Staphylococcus aureus Jumbo phage vB_StaM_SA1 | FINGFJCE_00174 | 95% | 1.00E-95 | 49.68% | 305 |
| 17 | Lys84 | Natural lysin | Staphylococcus aureus phage qdsa002 | IBPELELM_00072 | 100% | 0 | 99.80% | 495 |
| 18 | LysA72 | Natural lysin | Staphylococcus aureus phage phiIPLA35 | AHJFMBFC_00060 | 100% | 0 | 100.00% | 484 |
| 19 | LysB4EAD-LysSA11 | Chimeric lysin | LysB4 CHAP + LysSA11 | FINGFJCE_00175 | 58% | 0 | 100.00% | 252 |
| 20 | LysB4-LysSA11 | Chimeric lysin | LysB4 + LysSA11 | FINGFJCE_00175 | 49% | 0 | 100.00% | 252 |
| 21 | LysC | Natural lysin | Clostridium intestinale URNW | No significant similarity found. | | | | |
| 22 | LysC1C | Natural lysin | Staphylococcus aureus phage phiIPLA-C1C | IONBAAPO_00194 | 100% | 0 | 100.00% | 484 |
| 23 | LysCSA13 | Natural lysin | Staphylococcus aureus phage CSA13 | JKHLCIIN_00007 | 100% | 0 | 100.00% | 249 |
| 24 | Lysdb | Natural lysin | Lactobacillus delbrueckii phage phiLdb | GPCBFABN_00023 | 54% | 1.00E-05 | 24.24% | 272 |
| 25 | LysDZ25 | Natural lysin | Staphylococcus aureus phage DZ25 | HJGJHIOB_00042 | 100% | 0 | 96.07% | 484 |
| 26 | LysF1 | Natural lysin | Staphylococcus phage K1/420 | GNAFGFLF_00055 | 100% | 0 | 100.00% | 495 |
| 27 | LysGH15 | Natural lysin | Staphylococcus aureus phage GH15 | IBPELELM_00072 | 100% | 0 | 100.00% | 495 |
| 28 | LysH5 | Natural lysin | Staphylococcus aureus phage ΦH5 | HDKPDOPD_00062 | 98% | 0 | 100.00% | 481 |
| 29 | LysK | Natural lysin | Staphylococcus aureus phage K | GNAFGFLF_00055 | 100% | 0 | 100.00% | 495 |
| 30 | LySMP | Natural lysin | Streptococcus suis phage SMP | No significant similarity found. | | | | |
| 31 | LysMR-5 | Natural lysin | Staphylococcus aureus phage MR-5 | GNAFGFLF_00055 | 100% | 0 | 99.19% | 495 |
| 32 | LysP108 | Natural lysin | Staphylococcus aureus phage P108 | IBPELELM_00072 | 100% | 0 | 99.66% | 495 |
| 33 | LysPhi11 | Natural lysin | Staphylococcus aureus phage phi11 | NFCHLNFF_00064 | 100% | 0 | 100.00% | 481 |
| 34 | LysPhi80 | Natural lysin | Staphylococcus aureus phage phi80 | LGIGLCNC_00021 | 100% | 0 | 100.00% | 484 |
| 35 | Lys-phiSA012 | Natural lysin | Staphylococcus aureus phage phiSA012 | KOOKAECI_00053 | 100% | 0 | 100.00% | 495 |
| 36 | LysRODI | Natural lysin | Staphylococcus aureus phage phiIPLA-RODI | AIKLEEOI_00058 | 100% | 0 | 100.00% | 496 |
| 37 | LysSA11 | Natural lysin | Staphylococcus aureus phage SA11 | FINGFJCE_00175 | 100% | 0 | 100.00% | 252 |
| 38 | LysSA11-LysB4 | Chimeric lysin | LysSA11 + LysB4 | FINGFJCE_00175 | 49% | 0 | 100.00% | 252 |
| 39 | LysSA11-LysB4EAD | Chimeric lysin | LysSA11 + LysB4 CHAP | FINGFJCE_00175 | 58% | 0 | 100.00% | 252 |
| 40 | LysSA12 | Natural lysin | Staphylococcus aureus phage SA12 | IPFAMKEO_00058 | 100% | 0 | 100.00% | 481 |
| 41 | LysSAP27 | Natural lysin | Staphylococcus aureus phage vB_SauS-SAP27 | DAKCPLAH_00046 | 100% | 0 | 100.00% | 481 |
| 42 | LysSAP33 | Natural lysin | Staphylococcus phage SAP33 | GCF_000204665_1_pp2:R:2531-3286 | 100% | 0 | 100.00% | 251 |
| 43 | LysSAP8 | Natural lysin | Staphylococcus aureus phage SAP8 | HJGJHIOB_00042 | 100% | 0 | 100.00% | 484 |
| 44 | LysSS | Natural lysin | Salmonella phage SS3e | JAOKKBPI_00227 | 60% | 1.00E-09 | 35.64% | 154 |
| 45 | LysWMY | Natural lysin | Staphylococcus warneri phage WMY | JNLHGAHF_00166 | 99% | 0 | 55.79% | 467 |
| 46 | LytSD | Natural lysin | Streptomyces avermitilis phage phiSASD1 | No significant similarity found. | | | | |
| 47 | MMPphg | Natural lysin | Meiothermus phage MMP17 | GCF_003609895_1_pp1:R:2707-8907 | 66% | 3.00E-06 | 28.85% | 2066 |
| 48 | P10N-V12C | Chimeric lysin | LysEF-P10 CHAP + PlyV12 SH3b | POOPNMAA_00067 | 46% | 2.00E-19 | 35.48% | 629 |
| 49 | P28 | Natural lysin | Stenotrophomonas maltophilia P28 | FHAKCLKA_02057 | 27% | 2.00E-05 | 47.92% | 231 |
| 50 | P9ly | Natural lysin | Shigella phage PSD9 | JAOKKBPI_00227 | 91% | 4.00E-04 | 26.49% | 154 |
| 51 | Phi11 | Natural lysin | Staphylococcus aureus phage Φ11 | NFCHLNFF_00064 | 100% | 0% | 91.79% | 481 |
| 52 | Ply17 | Natural lysin | Pseudomonas aeruginosa phage phiYY | IONBAAPO_00174 | 17% | 3.00E-04 | 48.78% | 220 |
| 53 | Ply2638 | Natural lysin | Staphylococcus aureus phage 2638A | BOGOAIDK_00021 | 100% | 0 | 100.00% | 486 |
| 54 | Ply700 | Natural lysin | Streptococcus uberis (ATCC 700407) prophage | GCF_024741575_1_pp2:F:24716-25636 | 24% | 0.003 | 30.43% | 306 |
| 55 | PlyARI | Natural lysin | Streptococcus suis phage PhiARI0460-1 | JANNAGLN_00024 | 60% | 5.00E-18 | 36.49% | 639 |
| 56 | PlyGRCS | Natural lysin | Staphylococcus aureus phage GRCS | ONMOJPPK_00014 | 100% | 0 | 100.00% | 250 |
| 57 | Plypy | Natural lysin | Streptococcus pyogenes prophage | GNHENCMH_00049 | 79% | 4.00E-15 | 29.06% | 645 |
| 58 | PlyTW | Natural lysin | Staphylococcus aureus phage Twort | JNLHGAHF_00166 | 100% | 0 | 100.00% | 467 |
| 59 | PlyV12 | Natural lysin | Enterococcus faecalis phage Φ1 | BPOCJEBH_00086 | 19% | 6.00E-09 | 45.83% | 356 |
| 60 | S25-3 | Natural lysin | Staphylococcus phage S25-3 | AOFILPCH_00077 | 100% | 0 | 100.00% | 495 |
| 61 | SA.100 | Chimeric lysin | Lysostaphin M23 + Ply2638 amidase+Ply2638 SH3b | BOGOAIDK_00021 | 97% | 0 | 77.62% | 486 |
| 62 | SAL-1 | Natural lysin | Staphylococcus aureus phage SAP-1 | IBPELELM_00072 | 100% | 0 | 99.80% | 495 |
| 63 | SAL-2 | Natural lysin | Staphylococcus aureus phage SAP-2 | AEDHGBPN_00014 | 100% | 0 | 100.00% | 249 |
| 64 | SH2lysin | Natural lysin | Staphylococcus haemolyticus prophage ΦSH2 | DENIKHOF_00001 | 99% | 0 | 66.53% | 487 |
| 65 | TSPphg | Natural lysin | Thermus phage TSP4 | AFMKMADN_00051 | 55% | 0.014 | 31.18% | 478 |
| 66 | VAH88 | Natural lysin | Staphylococcus aureus phage 88 | EBKFGABG_00023 | 100% | 0 | 99.84% | 624 |
| 67 | XZ.700 | Chimeric lysin | Lysostaphin M23 + Ply2638 amidase+Ply2638 SH3b | BOGOAIDK_00021 | 96% | 0 | 75.54% | 486 |

**Table S3.** Natural lysins for experimental verification.

| **Natural lysins** | **Accession** | **Domain Construction** |
| --- | --- | --- |
| LLysSA16 | JGCGENHE_00167 | cl00222 |
| LLysSA17 | GCF_014334515_1_pp1_F_51933-52937 | PF01476, PF01476, PF01476, PF05257 |
| LLysSA18 | IMEFFGBA_00072 | cl02713 |
| LLysSA19 | BPOCJEBH_00086 | PF01520, cl17036 |
| LLysSA20 | GCF_022494545_1_pp3_R_1-1371 | PF01551 |
| LLysSA22 | GCF_003945425_1_pp2_R_1-1887 | cl23994, PF13472 |
| LLysSA23 | GCF_001019395_2_pp1_R_3683-5266 | PF18994, cl23994, cl23994 |

**Table S4.** Chimeric lysins for experimental verification.

| **Chimeric lysins** | **EAD** | | **CBD** | | |
| --- | --- | --- | --- | --- | --- |
|  | **Accession** | **Domain Construction** | | **Accession** | **Domain Construction** |
| LLysSA8 | HDKPDOPD_00058 | PF05257 | | CF-301 | PF08460 |
| LLysSA24 | GCF_000009645_1_pp1:R:5425-9954 | PF01551 | | CF-301 | PF08460 |
| LLysSA25 | GCF_000009645_1_pp1:R:5425-9954 | PF01551 | | GCF_014334515_1_pp1_F_51933-52937 | PF01476 |
| LLysSA26 | GCF_000009645_1_pp1:R:5425-9954 | PF01551 | | GCF_000144955_2_pp2:R:1482-5993 | PF10145 |
| LLysSA9 | EAONIOPL_00076 | PF05257 | | CF-301 | PF08460 |
| LLysSA27 | EAONIOPL_00076 | PF05257 | | GCF_014334515_1_pp1_F_51933-52937 | PF01476 |
| LLysSA28 | EAONIOPL_00076 | PF05257 | | GCF_000144955_2_pp2:R:1482-5993 | PF10145 |
| LLysSA29 | FINGFJCE_00175 | PF05257 | | CF-301 | PF08460 |
| LLysSA30 | FINGFJCE_00175 | PF05257 | | GCF_014334515_1_pp1_F_51933-52937 | PF01476 |
| LLysSA31 | FINGFJCE_00175 | PF05257 | | GCF_000144955_2_pp2:R:1482-5993 | PF10145 |

**Table S5.** Bacterial strains used in this study.

| **Species** | **Strain** | **Note** |
| --- | --- | --- |
| *Staphylococcus aureus* | LSA139 | ATCC25923 |
|  | LSA140 | ATCC6538 |
|  | LSA141 | ATCC8095 |
|  | LSA142 | RN4220 |
|  | LSA144 | ATCC29213 |
|  | LSA775 | Lab stock |
|  | LSA795 | Lab stock |
|  | LSA1565 | USA300 |
|  | LSA1566 | ATCC43300 |
| *Staphylococcus epidermidis* | LSEP1 | Lab stock |
|  | LSEP2 | Lab stock |
| *Staphylococcus hominis* | LSHO2 | Lab stock |
|  | LSHO3 | Lab stock |
| *Staphylococcus haemolyticus* | LSHA1 | Lab stock |
|  | LSHA2 | Lab stock |
| *Staphylococcus capitis* | LSCA1 | Lab stock |
|  | LSCA2 | Lab stock |
| *Staphylococcus saprophyticus* | LSSA1 | Lab stock |
| *Staphylococcus caprae* | LSCAP1 | Lab stock |
| *Staphylococcus pseudintermedius* | LSPS1 | Lab stock |
| *Streptococcus suis* | SC19 | Lab stock |
|  | LSM122 | Lab stock |
| *Acinetobacter baumannii* | LAB1 | Lab stock |
|  | LAB2 | Lab stock |
|  | LAB3 | Lab stock |
| *Pseudomonas aeruginosa* | LPA8 | Lab stock |
|  | LPA9 | Lab stock |
|  | LPA10 | Lab stock |
| *Klebsiella pneumoniae* | LKP11 | Lab stock |
|  | LKP397 | Lab stock |
|  | LKP504 | Lab stock |
| *Escherichia coli* | LEC30 | Lab stock |
|  | LEC32 | ATCC 18683 |
|  | LEC33 | ATCC HG15 |

**Table S6.** The minimum inhibitory concentration of LLysSA9 against 132 tested *Staphylococcus aureus*.

| **Strain** | **Type** | **Sequence Type** | **MIC**  **(μg/mL)** | **Strain** | **Type** | **Sequence Type** | **MIC**  **(μg/mL)** |
| --- | --- | --- | --- | --- | --- | --- | --- |
| LSA745 | MRSA | 1 | 0.5 | LSA139 | MSSA | 243 | 1 |
| LSA754 | MRSA | 1 | 1 | LSA642 | MRSA | 338 | 0.5 |
| LSA985 | MRSA | 1 | 2 | LSA550 | MRSA | 338 | 2 |
| LSA225 | MRSA | 1 | 1 | LSA592 | MRSA | 398 | 2 |
| LSA460 | MSSA | 1 | 2 | LSA795 | MRSA | 398 | 2 |
| LSA522 | MRSA | 5 | 0.5 | LSA234 | MSSA | 398 | 4 |
| LSA866 | MSSA | 5 | 0.5 | LSA244 | MSSA | 398 | 32 |
| LSA143 | MSSA | 5 | 1 | LSA324 | MSSA | 398 | 8 |
| LSA144 | MSSA | 5 | 1 | LSA383 | MSSA | 398 | 4 |
| LSA240 | MSSA | 5 | 2 | LSA393 | MSSA | 398 | 2 |
| LSA242 | MSSA | 5 | 32 | LSA487 | MSSA | 398 | 4 |
| LSA245 | MSSA | 5 | 4 | LSA140 | MSSA | 464 | 0.5 |
| LSA248 | MSSA | 5 | 4 | LSA691 | MRSA | 508 | 1 |
| LSA428 | MSSA | 5 | 4 | LSA691 | MRSA | 508 | 1 |
| LSA684 | MSSA | 6 | 0.5 | LSA560 | MRSA | 509 | 1 |
| LSA237 | MSSA | 6 | 4 | LSA1469 | MSSA | 522 | 1 |
| LSA239 | MSSA | 6 | 2 | LSA628 | MSSA | 623 | 0.5 |
| LSA387 | MSSA | 6 | 4 | LSA715 | MRSA | 630 | 0.25 |
| LSA425 | MSSA | 6 | 4 | LSA541 | MRSA | 630 | 1 |
| LSA450 | MSSA | 6 | 2 | LSA555 | MSSA | 630 | 0.5 |
| LSA582 | MSSA | 7 | 1 | LSA623 | MSSA | 672 | 0.25 |
| LSA235 | MSSA | 7 | 2 | LSA681 | MSSA | 944 | 0.25 |
| LSA236 | MSSA | 7 | 2 | LSA338 | MSSA | 950 | 8 |
| LSA246 | MSSA | 7 | 2 | LSA698 | MRSA | 965 | 1 |
| LSA337 | MSSA | 7 | 4 | LSA249 | MSSA | 965 | 2 |
| LSA339 | MSSA | 7 | 4 | LSA968 | MRSA | 968 | 2 |
| LSA424 | MSSA | 7 | 4 | LSA587 | MSSA | 1281 | 0.5 |
| LSA444 | MSSA | 7 | 1 | LSA816 | MSSA | 1281 | 0.5 |
| LSA546 | MRSA | 8 | 2 | LSA452 | MSSA | 1281 | 2 |
| LSA141 | MSSA | 8 | 2 | LSA775 | MRSA | 1376 | 1 |
| LSA142 | MSSA | 8 | 1 | LSA784 | MRSA | 1376 | 1 |
| LSA1565 | MRSA | 8 | 2 | LSA747 | MRSA | 1821 | 0.5 |
| LSA785 | MRSA | 9 | 0.25 | LSA462 | MSSA | 1920 | 4 |
| LSA851 | MRSA | 9 | 0.25 | LSA464 | MSSA | 1920 | 4 |
| LSA1050 | MRSA | 9 | 1 | LSA662 | MSSA | 2114 | 1 |
| LSA578 | MSSA | 15 | 0.5 | LSA1474 | MSSA | 3444 | 2 |
| LSA463 | MSSA | 15 | 8 | LSA1477 | MSSA | 3444 | 4 |
| LSA661 | MRSA | 22 | 1 | LSA531 | MRSA | 4513 | 0.5 |
| LSA580 | MSSA | 22 | 0.5 | LSA1528 | MSSA | 6262 | 0.5 |
| LSA429 | MSSA | 22 | 4 | LSA1529 | MSSA | 6262 | 1 |
| LSA440 | MSSA | 22 | 2 | LSA1526 | MSSA | 6263 | 0.125 |
| LSA481 | MSSA | 22 | 4 | LSA1527 | MSSA | 6263 | 0.5 |
| LSA482 | MSSA | 22 | 2 | LSA1510 | MSSA | 6264 | 1 |
| LSA494 | MSSA | 22 | 4 | LSA1471 | MSSA | 6265 | 2 |
| LSA612 | MSSA | 25 | 0.25 | LSA1508 | MSSA | 6265 | 2 |
| LSA445 | MSSA | 25 | 2 | LSA1465 | MSSA | 6266 | 0.5 |
| LSA711 | MRSA | 30 | 4 | LSA1475 | MSSA | 6266 | 0.5 |
| LSA476 | MSSA | 30 | 2 | LSA1462 | MSSA | 6267 | 0.5 |
| LSA1566 | MRSA | 39 | 4 | LSA1463 | MSSA | 6267 | 8 |
| LSA605 | MRSA | 45 | 1 | LSA637 | MSSA | 6543 | 0.5 |
| LSA608 | MRSA | 45 | 1 | LSA666 | MSSA | 6544 | 0.5 |
| LSA599 | MRSA | 59 | 0.25 | LSA624 | MSSA | 6545 | 0.5 |
| LSA706 | MRSA | 59 | 0.25 | LSA668 | MRSA | 6546 | 2 |
| LSA625 | MRSA | 59 | 1 | LSA543 | MRSA | 6547 | 0.5 |
| LSA660 | MRSA | 59 | 4 | LSA561 | MRSA | 6548 | 1 |
| LSA753 | MRSA | 59 | 16 | LSA675 | MRSA | 6549 | 2 |
| LSA227 | MRSA | 59 | 1 | LSA748 | MRSA | 6550 | 1 |
| LSA535 | MRSA | 88 | 2 | LSA601 | MRSA | 6551 | 0.25 |
| LSA506 | MRSA | 121 | 0.25 | LSA651 | MSSA | 6552 | 0.5 |
| LSA534 | MRSA | 121 | 2 | LSA1011 | MRSA | 6554 | 0.5 |
| LSA583 | MSSA | 188 | 0.5 | LSA1012 | MRSA | 6554 | 0.5 |
| LSA477 | MSSA | 188 | 2 | LSA997 | MRSA | 6555 | 0.125 |
| LSA513 | MRSA | 239 | 0.5 | LSA894 | MRSA | 6556 | 0.25 |
| LSA710 | MRSA | 239 | 2 | LSA443 | MSSA | new | 1 |
| LSA726 | MRSA | 239 | 2 | LSA421 | MSSA | / | 4 |
| LSA727 | MRSA | 239 | 16 | LSA455 | MSSA | / | 4 |

**Table S7.** The running time of DeepLysin required to mine putative lysins from the phage genomes, and the number of putative lysins obtained.

| **Target** | **The number of phage genomes** | **Time** | **The number of putative lysins** | **The number of putative lysins with estimated antibacterial activity > 0.5** |
| --- | --- | --- | --- | --- |
| *E. coli* | 405 | 1h 1min 27s | 511 | 358 |
| *S. aureus* | 189 | 20min 17s | 345 | 337 |
| *K. pneumoniae* | 136 | 23min 52s | 71 | 49 |
| *A. baumannii* | 65 | 15min 23s | 77 | 59 |
| *P. aeruginosa* | 253 | 31min 38s | 79 | 75 |
| *E. faecium* | 53 | 17min 23s | 47 | 23 |

**Table S8.** The running time of DeepLysin required to mine putative lysins from the bacterial genomes, and the number of putative lysins obtained.

| **Target** | **The number of bacterial genomes** | **Time** | **The number of putative lysins** | **The number of putative lysins with estimated antibacterial activity > 0.5** |
| --- | --- | --- | --- | --- |
| *E. coli* | 405 | 58h 3min 18s | 173 | 123 |
| *S. aureus* | 189 | 52h 3min 19s | 150 | 150 |
| *K. pneumoniae* | 136 | 58h 23min 10s | 198 | 137 |
| *A. baumannii* | 65 | 37h 44min 49s | 106 | 95 |
| *P. aeruginosa* | 253 | 68h 59min 23s | 231 | 163 |
| *E. faecium* | 53 | 17h 50min 55s | 58 | 33 |

**Table S9.** Published domains of lysin collected from literatures.

| **Domain** | **Domain Cluster Name** | **Accession** | **Domain** | **Domain Cluster Name** | **Accession** | **Domain** | **Domain Cluster Name** | **Accession** |
| --- | --- | --- | --- | --- | --- | --- | --- | --- |
| EAD | Glyco_hydro_19 | PF00182 | CBD | Amidase02_C | PF12123/cl13548 | Unknown | His_biosynth | PF00977 |
|  | NLPC_P60 | PF00877 |  | Big_2 | PF02368 |  | HH_signal | PF01085 |
|  | Transglycosylase | PF00912 |  | CW_7/Cpl-7 | PF08230 |  | CSD | PF00313 |
|  | Phage_lysozyme | PF00959 |  | CW_binding_1 | PF01473 |  | NHase_beta | PF02211 |
|  | Glyco_hydro_25 | PF01183/cl10448 |  | CW_binding_2 | PF04122 |  | DUF1161 | PF06649 |
|  | Peptidase_M15 | PF01427 |  | DUF3597 | PF12200 |  | Extensin-like_C | PF06904 |
|  | SLT | PF01464/cl00222 |  | LGFP | PF08310 |  | D123 | PF07065 |
|  | Amidase_2 | PF01510 |  | LysM | PF01476 |  | Gmx_para_CXXCG | PF09535 |
|  | Amidase_3 | PF01520/cl02713 |  | PG_binding_1 | PF01471 |  | DUF2099 | PF09872 |
|  | Peptidase_M23 | PF01551 |  | PG_binding_3 | PF09374 |  | PhageMin_Tail | PF10145 |
|  | Glucosaminidase | PF01832 |  | PSA_CBD | PF18341 |  | TcpQ | PF10671 |
|  | VanY | PF02557 |  | SH3_3 | PF08239 |  | DUF2514 | PF10721 |
|  | YkuD | PF03734 |  | SH3_5 | PF08460/cl17036 |  | ERAP1_C | PF11838 |
|  | CHAP | PF05257 |  | SPOR | PF05036 |  | HTH_Tnp_IS1 | PF12759 |
|  | Amidase_5 | PF05382 |  | ZoocinA_TRD | PF16775 |  | Hint_2 | PF13403 |
|  | Glyco_hydro_108 | PF05838 |  | Gp5_OB | PF06714 |  | Kelch_4 | PF13418 |
|  | Peptidase_M15_2 | PF05951 |  | Gp5_C | PF06715 |  | Lipase_GDSL_2 | PF13472 |
|  | Peptidase_C93 | PF06035 |  | ChW | PF07538 |  | DUF4407 | PF14362 |
|  | Transglycosylase | PF06737 |  | SLAP | PF03217 |  | BtrH_N | PF14399 |
|  | Hydrolase_2 | PF07486 |  | SLH | PF00395 |  | LAGLIDADG_3 | PF14528 |
|  | Peptidase_M15_3 | PF08291 |  | LCCL | PF03815 |  | Intein_splicing | PF14890 |
|  | Methyltransf_16 | PF10294 |  | PG_binding_2 | PF08823 |  | DUF4691 | PF15762 |
|  | Peptidase_U40 | PF10464 |  | SH3_4 | PF06347 |  | Na_Ca_ex_C | PF16494 |
|  | Glyco_hydro_cc | PF11790 |  |  |  |  |  |  |
|  | Muramidase | PF11860 |  |  |  |  |  |  |
|  | Peptidase_C70 | PF12385 |  |  |  |  |  |  |
|  | Peptidase_C39_2 | PF13529 |  |  |  |  |  |  |
|  | Peptidase_M15_4 | PF13539 |  |  |  |  |  |  |
|  | Lysozyme_like | PF13702 |  |  |  |  |  |  |
|  | Phage_lysozyme2 | PF18013 |  |  |  |  |  |  |
|  | 3D | PF06725 |  |  |  |  |  |  |
|  | Cutinase | PF01083 |  |  |  |  |  |  |
|  | FSH1 | PF03959 |  |  |  |  |  |  |
|  | GPW_gp25 | PF04965 |  |  |  |  |  |  |
|  | PE-PPE | PF08237 |  |  |  |  |  |  |
|  | Pesticin | PF16754 |  |  |  |  |  |  |
|  | Prok-JAB | PF14464 |  |  |  |  |  |  |
|  | Prophage_tail | PF06605/cl23994 |  |  |  |  |  |  |
|  | DUF3335 | PF11814 |  |  |  |  |  |  |
|  | Melibiase_2 | PF16499 |  |  |  |  |  |  |
|  | SLT_2 | PF13406 |  |  |  |  |  |  |

**Table S10.** Overview of the six features.

| **Feature Category** | **Feature name** | **Abbreviation** | **Dimension** | **Notes** |
| --- | --- | --- | --- | --- |
| Composition-based feature | Amino acid composition | AAC | 60 | Represented by the fraction of 20 different amino acids in full sequences. |
| Binary profile-based feature | Binary profiling feature | BPNC | 20 | One-hot encoding in proteins (Table S11). |
|  | Amino acid index feature | AAI | 36 | Hydrophobicity, hydrophilicity, steric parameter, solvation, hydropathy, amphiphilicity, size, polarity, hydrogen, net charge, molecular weight, residue volume properties, and full length of amino acids. |
|  | In grouped amino acid composition | GAAC | 15 | The 20 basic amino acids are categorized into five varieties based on their physicochemical properties (Table S12). |
| Position-based feature | Amino acid entropy | AAE | 60 | $AAE=\sum_{m+1}^{i=1} (\frac{s_{i}-s_{i-1}}{n}){log}_{2}(\frac{s_{i}-s_{i-1}}{n})$  where m represents the length of the protein M and n represents the number of the amino acid ‘A’ in the protein M, position of ‘A’ is marked as s_1_ and s_m_ in the sequence, and it is defined as s_0_=0 and s_m_+1=n+1. |
| Physicochemical property based feature | Composition-transition-distribution | CTD | 147 | The amino acid distribution patterns of a specific physicochemical property (Table S13). |

**Table S11.** Amino acids and their corresponding 20-dimensional binary vectors.

| **Amino acid** | **Vectory** |
| --- | --- |
| A | (1,0,0,0,0,0,0,0,0,0,0,0,0,0,0,0,0,0,0,0) |
| R | (0,1,0,0,0,0,0,0,0,0,0,0,0,0,0,0,0,0,0,0) |
| N | (0,0,1,0,0,0,0,0,0,0,0,0,0,0,0,0,0,0,0,0) |
| D | (0,0,0,1,0,0,0,0,0,0,0,0,0,0,0,0,0,0,0,0) |
| C | (0,0,0,0,1,0,0,0,0,0,0,0,0,0,0,0,0,0,0,0) |
| Q | (0,0,0,0,0,1,0,0,0,0,0,0,0,0,0,0,0,0,0,0) |
| E | (0,0,0,0,0,0,1,0,0,0,0,0,0,0,0,0,0,0,0,0) |
| G | (0,0,0,0,0,0,0,1,0,0,0,0,0,0,0,0,0,0,0,0) |
| H | (0,0,0,0,0,0,0,0,1,0,0,0,0,0,0,0,0,0,0,0) |
| I | (0,0,0,0,0,0,0,0,0,1,0,0,0,0,0,0,0,0,0,0) |
| L | (0,0,0,0,0,0,0,0,0,0,1,0,0,0,0,0,0,0,0,0) |
| K | (0,0,0,0,0,0,0,0,0,0,0,1,0,0,0,0,0,0,0,0) |
| M | (0,0,0,0,0,0,0,0,0,0,0,0,1,0,0,0,0,0,0,0) |
| F | (0,0,0,0,0,0,0,0,0,0,0,0,0,1,0,0,0,0,0,0) |
| P | (0,0,0,0,0,0,0,0,0,0,0,0,0,0,1,0,0,0,0,0) |
| S | (0,0,0,0,0,0,0,0,0,0,0,0,0,0,0,1,0,0,0,0) |
| T | (0,0,0,0,0,0,0,0,0,0,0,0,0,0,0,0,1,0,0,0) |
| W | (0,0,0,0,0,0,0,0,0,0,0,0,0,0,0,0,0,1,0,0) |
| Y | (0,0,0,0,0,0,0,0,0,0,0,0,0,0,0,0,0,0,1,0) |
| V | (0,0,0,0,0,0,0,0,0,0,0,0,0,0,0,0,0,0,0,1) |

**Table S12.** The category of 20 basic amino acids is based on their physicochemical properties.

| **Category** | **Amino acid** |
| --- | --- |
| aliphatic group | G, A, V, L, M, I |
| aromatic group | F, Y, W |
| positively charged group | K, R, H |
| negatively charged group | D, E |
| uncharged group | S, T, C, P, N, Q |

**Table S13.** Details for a group of CTD features according to specific physicochemical properties.

| Physicochemical property | Group 1 | Group 2 | Group 3 |
| --- | --- | --- | --- |
| Normalized Van der Waals volume | G, A, S, C, T, P, D | N, V, E, Q, I, L | M, H, K, F, R, Y, W |
| Charge | K, R | A, N, C, Q, G, H, I,  L, M, F, P, S, T, W, Y, V | D, E |
| Solvent accessibility | A, L, F, C, G, I, V, W | R, K, Q, E, N, D | M, P, S, T, H, Y |
| Polarizability | G, A, S, D, T | C, P, N, V, E, Q, I, L | K, M, H, F, R, Y, W |
| Polarity | L, I, F, W, C, M, V, Y | P, A, I, G, S | H, Q, R, K, N, E, D |
| Hydrophobicity | R, K, E, D, Q, N | G, A, S, T, P, H, T | C, V, I, L, M, F, W |
| Secondary structures | E, A, L, M, Q, K, R, H | V, I, Y, C, W, F, T | G, N, P, S, D |

**Supplementary Figures**


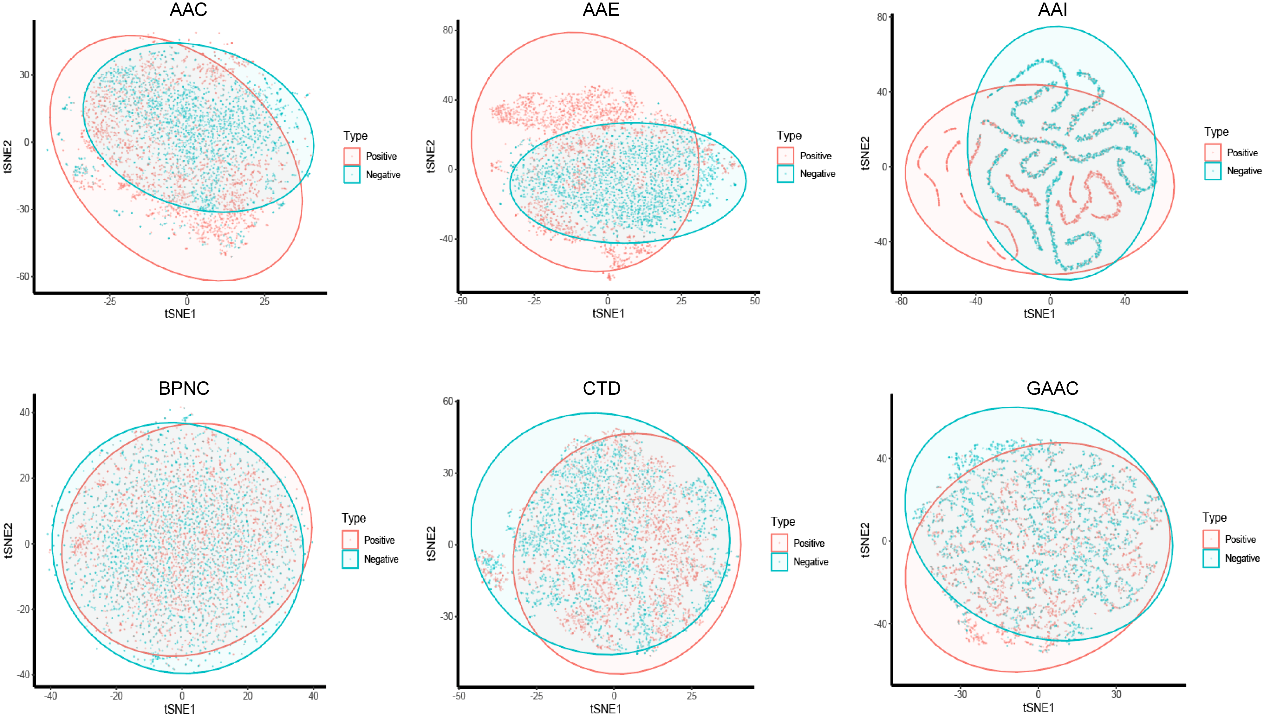


**Figure S1.** Dimensionality reduction visualization (tSNE) of individual metrics without calculation by the first layer of the stacking model.


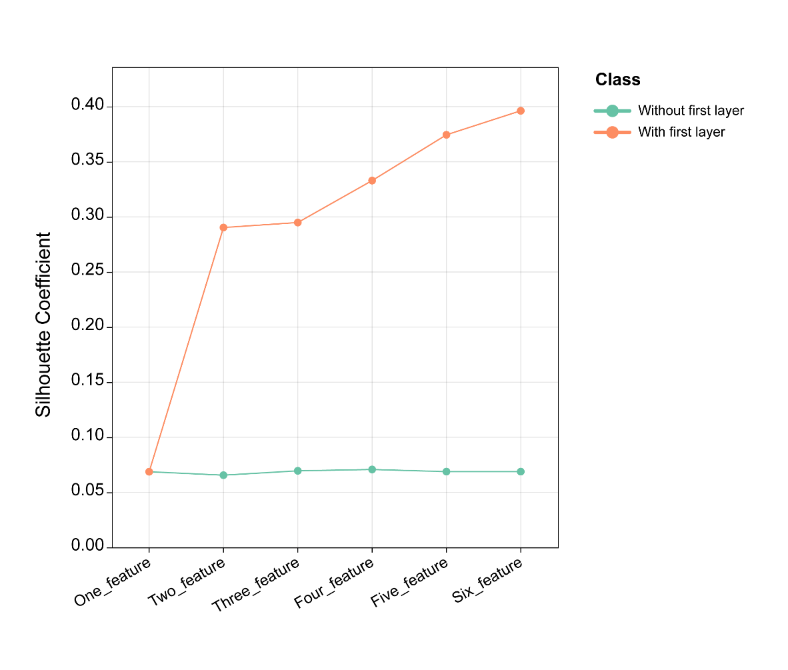


**Figure S2.** Silhouette Coefficient of different feature combinations. Orange represents that the Silhouette Coefficient was calculated with the first layer of stacking model, green indicates that it was calculated without the first layer of stacking model.


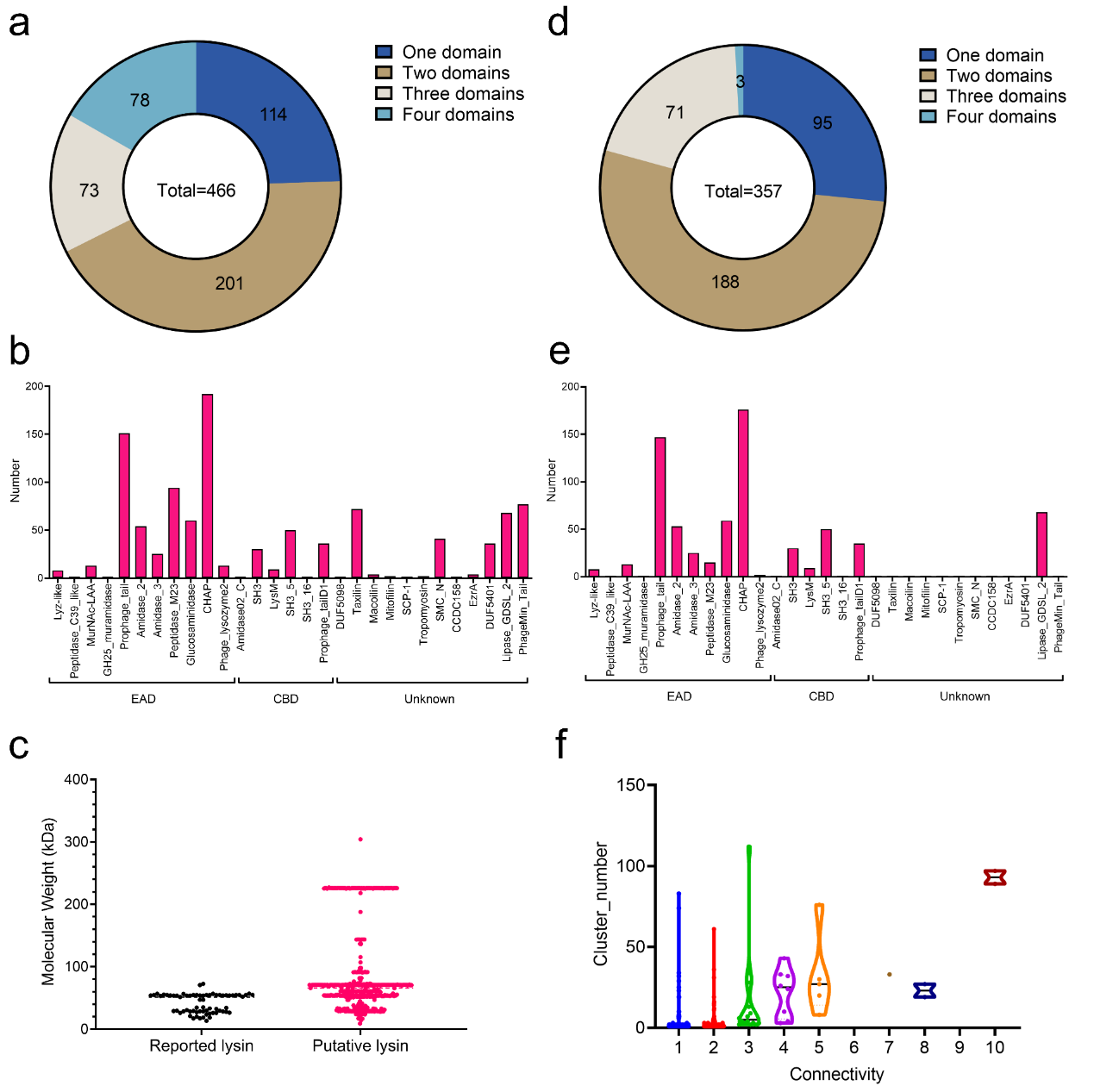


**Figure S3.** Analysis of *S. aureus* putative lysins. a) Distribution of the number of domains predicted per lysin (466 putative lysins, antibacterial activity > 0.5). Nearly 50% of the lysins harbored two predicted functional domains. b) Domain variability of 466 putative lysins. CHAP, Prophage_tail, and Peptidase_M23 were common enzymatically active domains, with SH3_5 being the preferred cell wall binding domain in *S. aureus* lysins. c) Molecular weight of reported lysins and putative lysins. d) Distribution of the number of domains predicted per lysin (357 putative lysins, MW < 80kDa). Consistent distribution was observed such that lysins possessing two domains took the dominant position. e) Domain variability of 357 putative lysins. The molecular weight threshold had a slight effect on the distribution of EAD and CBD, while greatly reducing the distribution of yet-undefined domains, indicating that empirical selection pre-filtered out some proteins unlikely to have antibacterial activity. f) The correlation between connectivity and cluster_number. The complete sequence of lysins was split according to domains. The split sequences with over 80% similarity were clustered as one node, and a total of 249 nodes (clusters) were formed. Connectivity means the possibility of a domain combining with other domains to form a complete lysin. Cluster_number means the number of sequences clustered by each node. There is no definite correlation between connectivity and cluster_number, but nodes with high connectivity tend to cover more similar sequences, meaning that this domain has a high propensity to cooperate with other domains to form complete lysins, and has adapted to the specific structure and characteristics of the host bacteria during natural evolution to efficiently regulate degradation of the peptidoglycan.


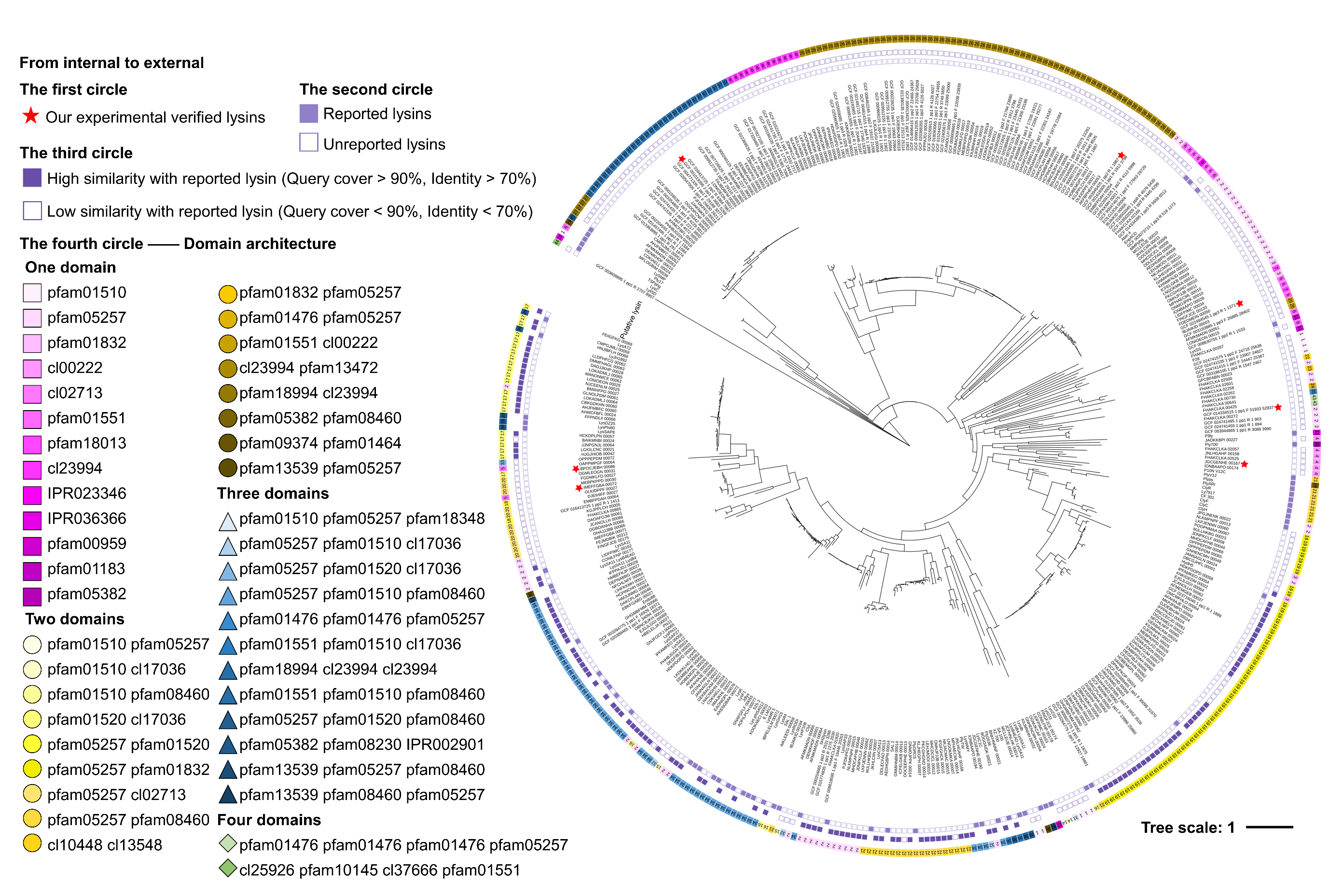


**Figure S4.** Selection of natural lysins. Larger version of Figure 3b.


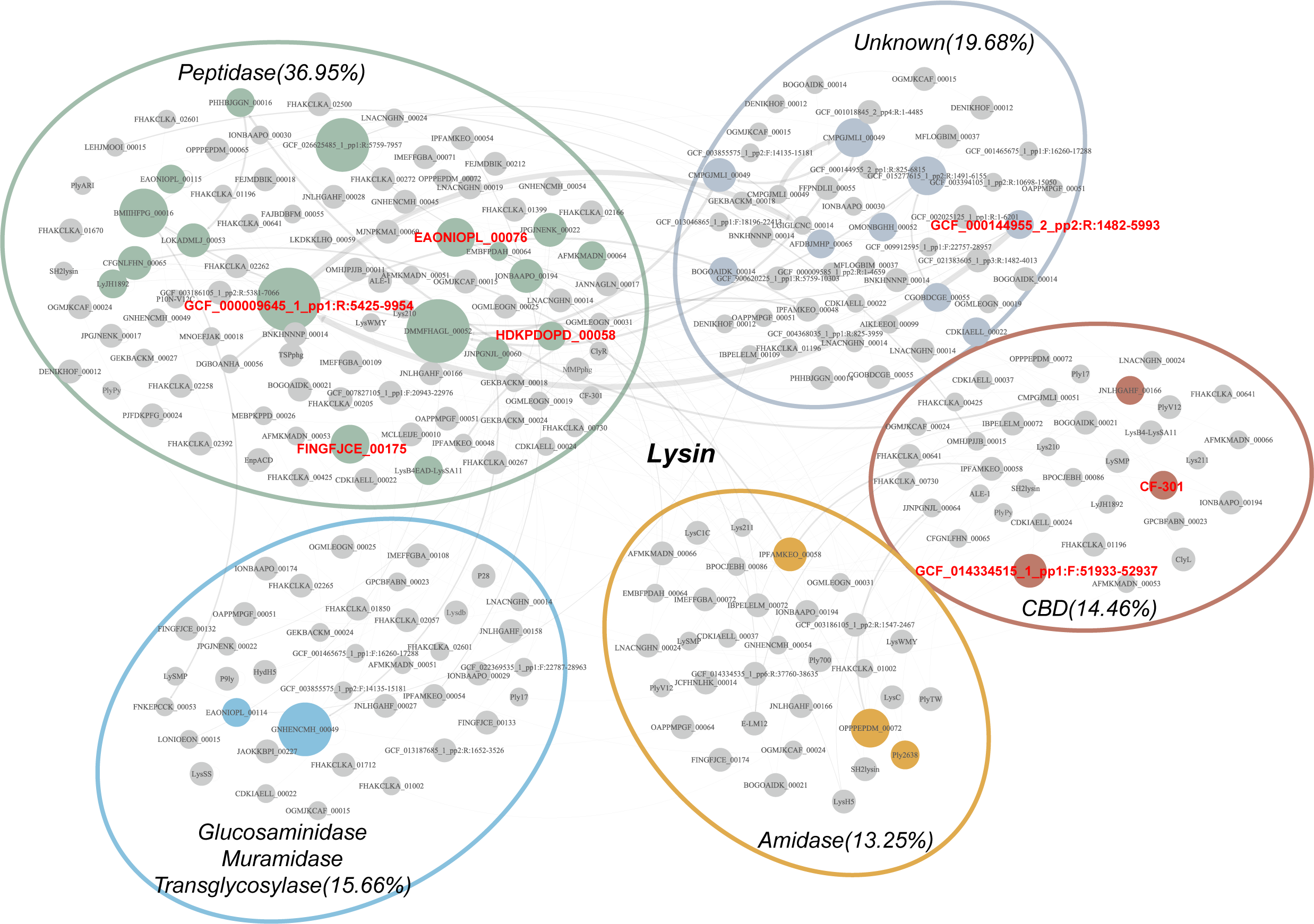


**Figure S5.** Selection of chimeric lysins. Larger version of Figure 3c.


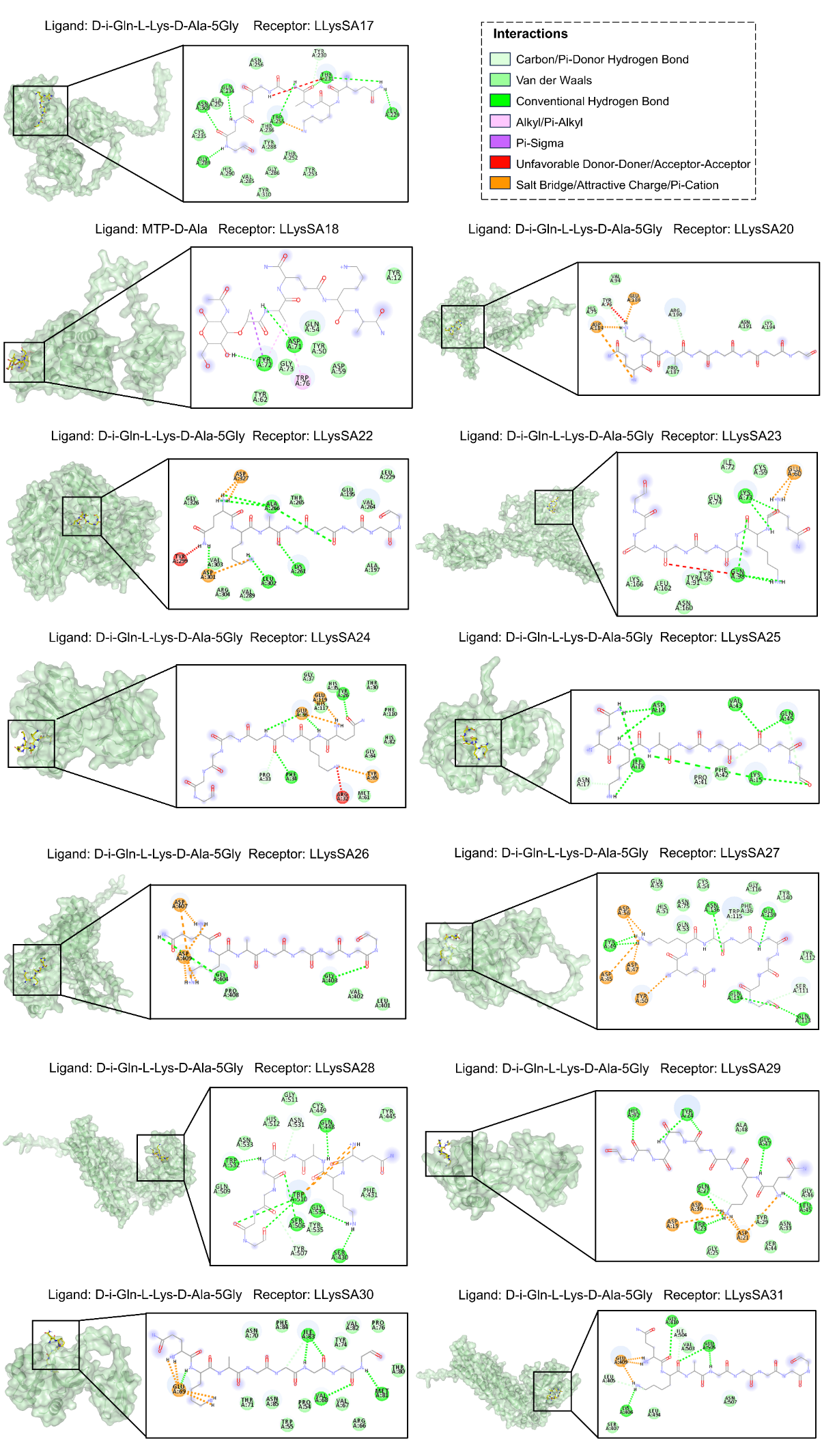


**Figure S6.** The results of molecular docking between lysin and peptidoglycan. The grooves on the surface of putative lysins (green) can accommodate peptidoglycan fragments (yellow), forming various interactions, indicating the possibility of interaction between lysins and peptidoglycan, as well as the possibility of antibacterial activity of lysins screened by DeepLysin.


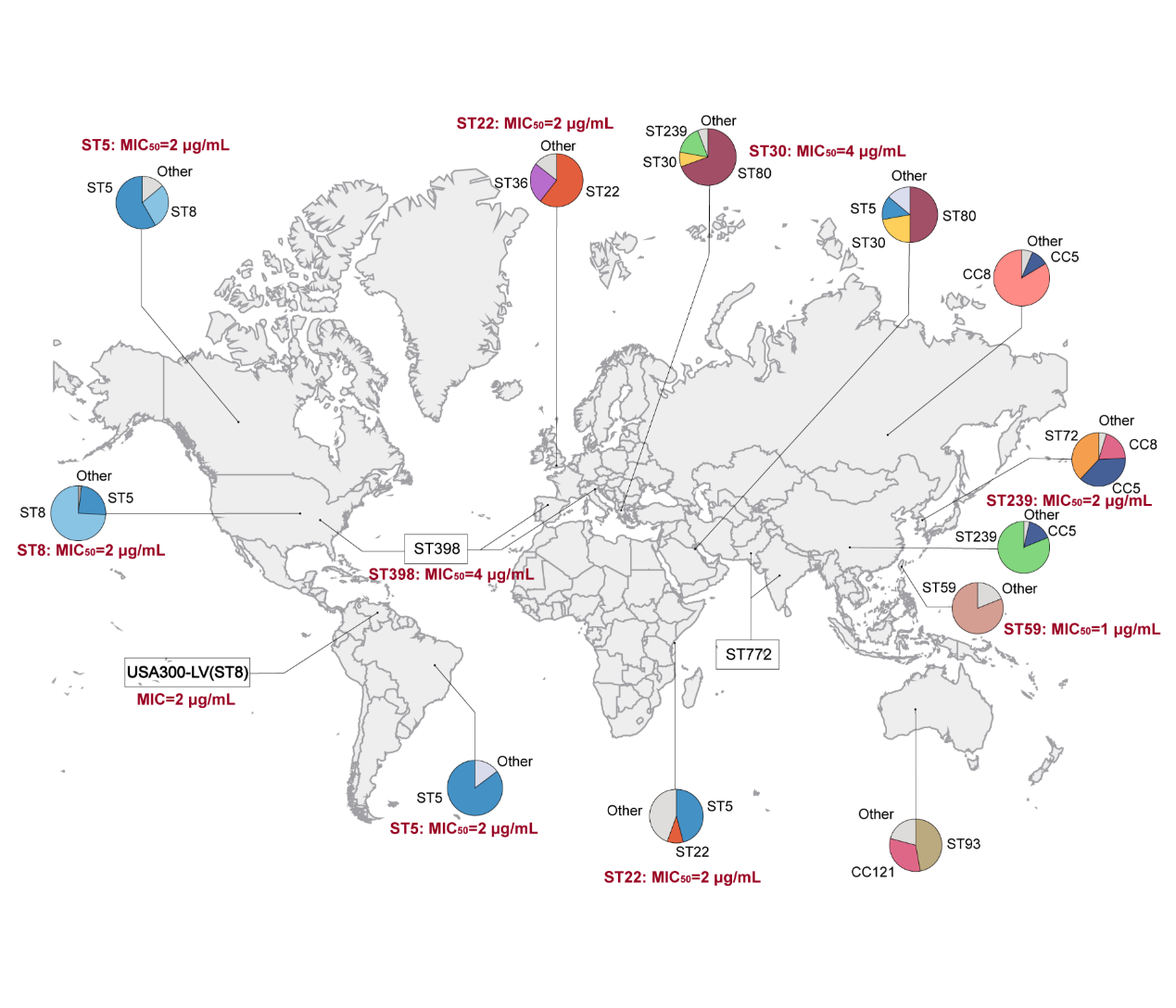


**Figure S7.** The global distribution of *Staphylococcus aureus* and the minimum inhibitory concentration of prevalent strain types in each region.


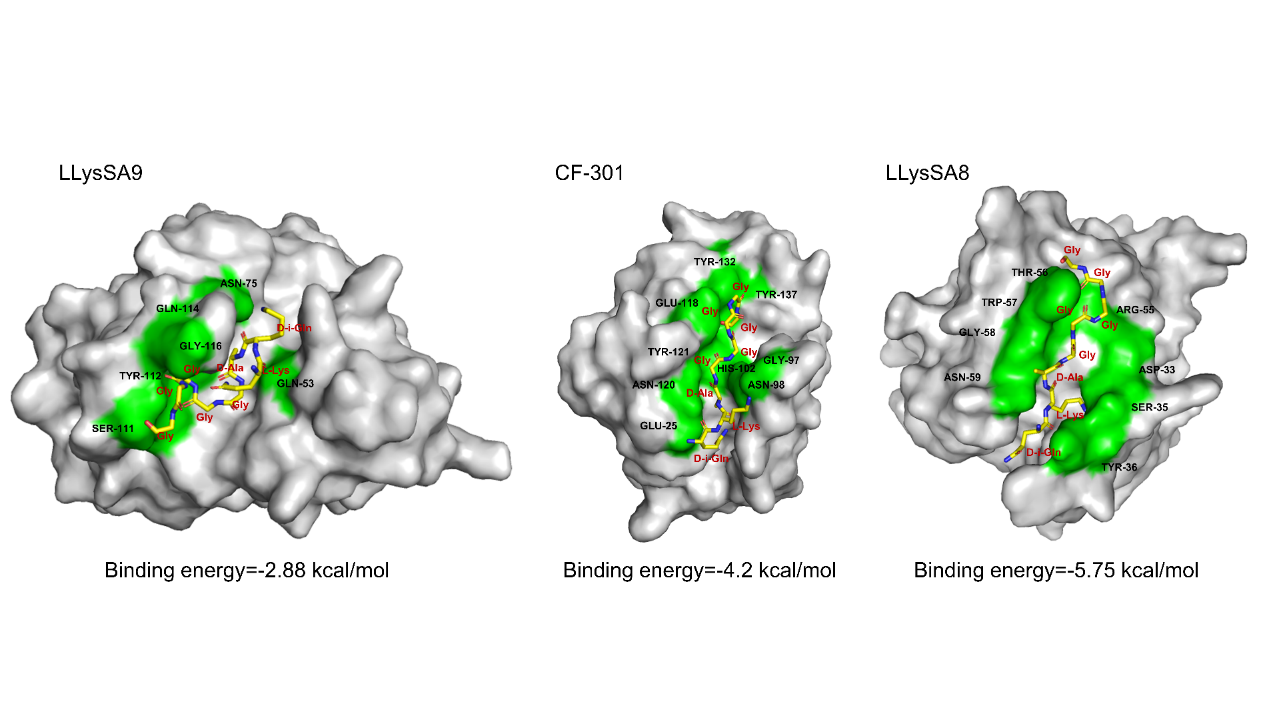


**Figure S8.** The binding energy between lysin domain and peptidoglycan fragment.


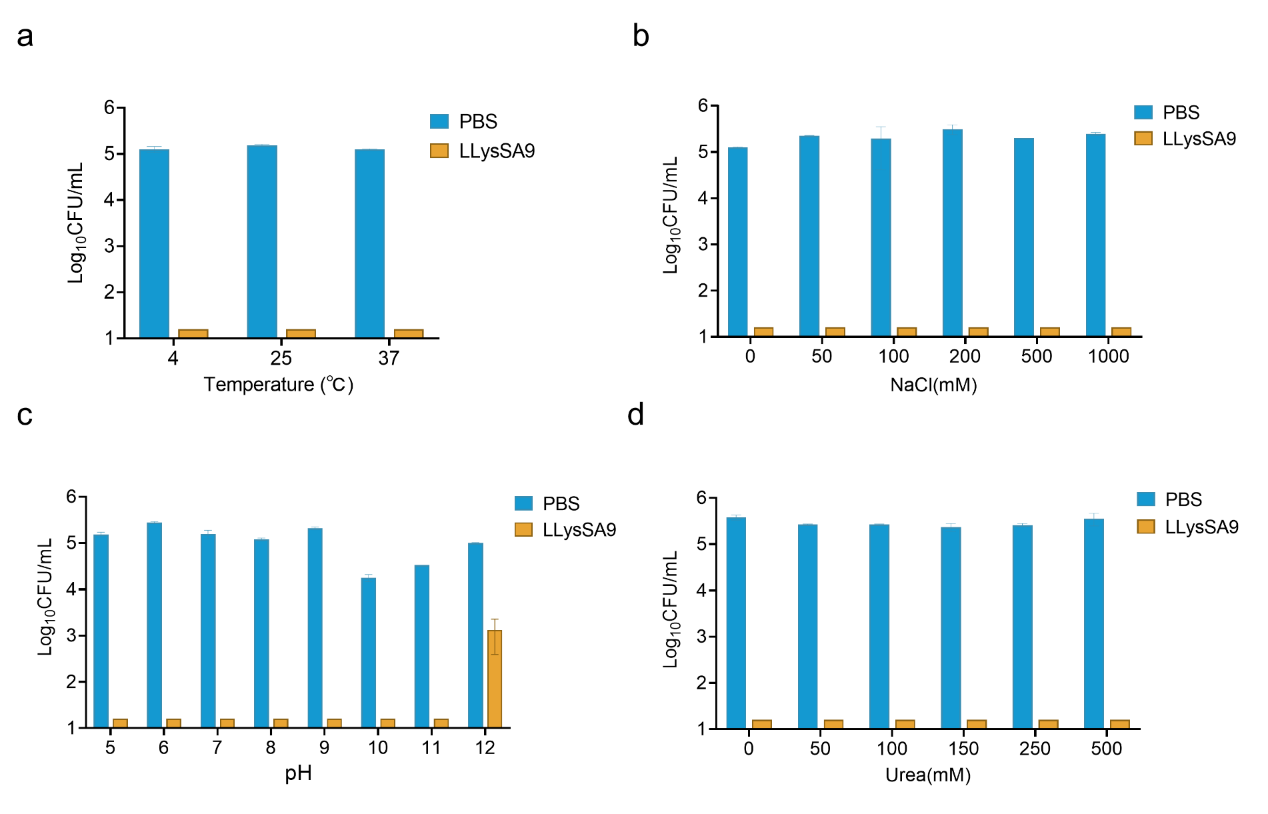


**Figure S9.** The antibacterial activity of LLysSA9 (50 μg/mL) against *Staphylococcus aureus* USA300 in different physiological conditions. a) Effect of temperature, b) effect of NaCl concentration, c) effect of pH, and d) effect of urea on the antibacterial activity of LLysSA9.


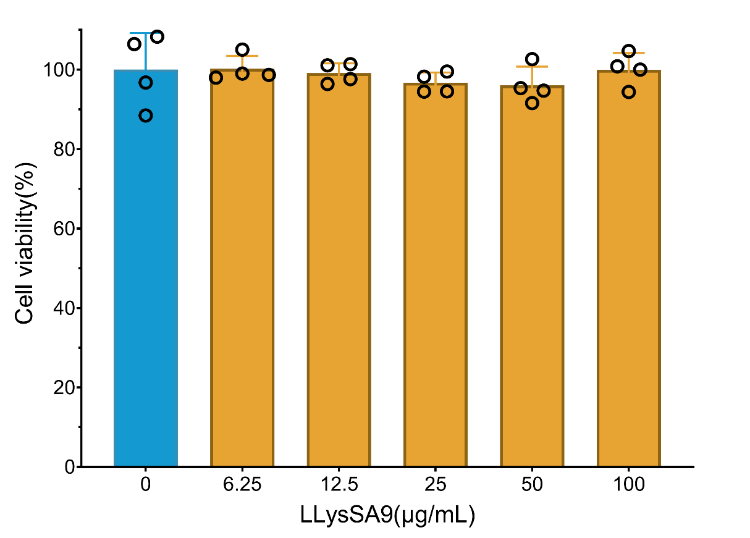


**Figure S10.** The safety of LLysSA9. Cytotoxicity activity of different concentrations of LLysSA9 on RAW264.7 cells.


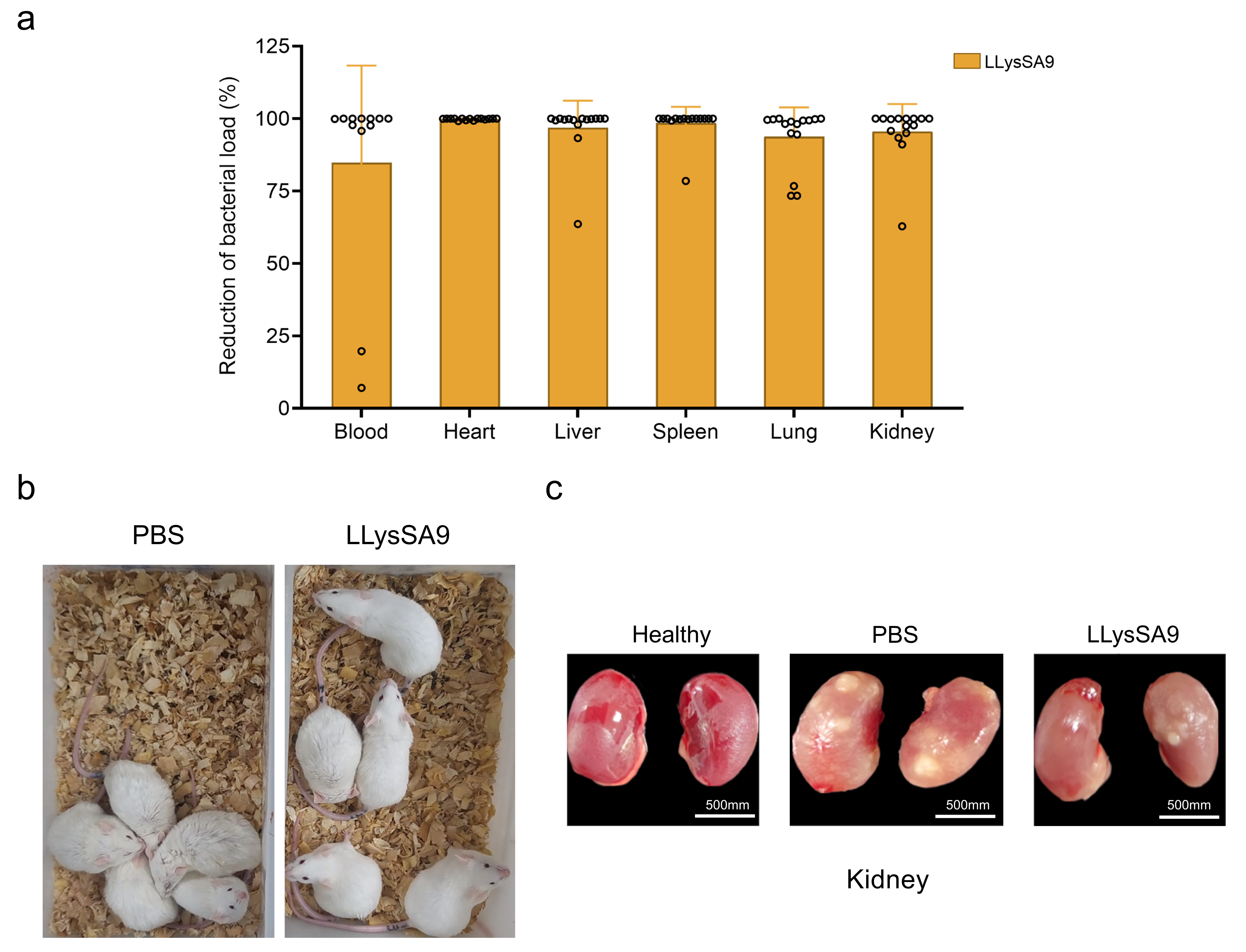


**Figure S11.** Therapeutic effect of LLysSA9 in a bacteremia mouse model. Mice were intravenously injected with *S. aureus* USA300 (2×10^8^ CFU/mouse) and 1.5 h later received a single intravenous dose of PBS buffer (controls) or 10 mg/kg LLysSA9. a) Reduction of bacterial load in blood and organs after 7 days of treatment or at the time of death. b) Clinical signs of mice. Control mice exhibited various clinical signs after bacterial challenge that were not observed in LLysSA9-treated mice, including decreased locomotor activity, piloerection, and flocking behavior. c) Pathological changes in mouse kidneys. The severe abscesses observed in the kidneys of control mice were dramatically attenuated with LLysSA9 treatment.


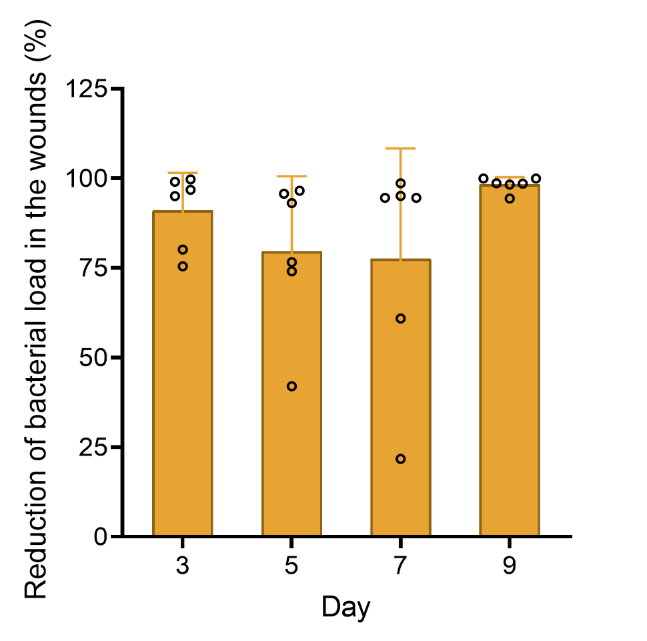


**Figure S12.** Efficacy of topical LLysSA9 in a skin wound infection mouse model. Each mouse received a 1-cm diameter round full‑thickness wound induced by punch, with *S. aureus* USA300 (5×10^8^ CFU/mouse) then injected subcutaneously around the wound. After 24 h, mice were treated topically on days 1, 3, 5, and 7 with either PBS (controls) or LLysSA9 (10 mg/kg). Mice were euthanized on day 9. Reduction of bacterial load in the wounds (n=6 mice per group).
